# Engineering with Biomedical Sciences Changing the Horizon of Healthcare-A Review

**DOI:** 10.1080/21655979.2024.2401269

**Published:** 2024-09-16

**Authors:** Induni N. Weerarathna, Praveen Kumar, Anurag Luharia, Gaurav Mishra

**Affiliations:** a School of Allied Health Sciences, Department of Biomedical Sciences, Jawaharlal Nehru Medical College, Datta Meghe Institute of Higher Education and Research, Wardha, Maharashtra, India; b Department of Computer Science and Medical Engineering, Datta Meghe Institute of Higher Education and Research, Wardha, Maharashtra, India; c Department of Radio Physicist and Radio Safety, Datta Meghe Institute of Higher Education and Research, Wardha, Maharashtra, India; d Department of Radio Diagnosis, Jawaharlal Nehru Medical College, Datta Meghe Institute of Higher Education and Research, Wardha, Maharashtra, India

**Keywords:** Biomedical science, biomedical engineering, biosensors, drug delivery systems, tissue engineering, robots, biomechanics, green biomaterials

## Abstract

In the dynamic realm of healthcare, the convergence of engineering and biomedical sciences has emerged as a pivotal frontier. In this review we go into specific areas of innovation, including medical imaging and diagnosis, developments in biomedical sensors, and drug delivery systems. Wearable biosensors, non-wearable biosensors, and biochips, which include gene chips, protein chips, and cell chips, are all included in the scope of the topic that pertains to biomedical sensors. Extensive research is conducted on drug delivery systems, spanning topics such as the integration of computer modeling, the optimization of drug formulations, and the design of delivery devices. Furthermore, the paper investigates intelligent drug delivery methods, which encompass stimuli-responsive systems such as temperature, redox, pH, light, enzyme, and magnetic responsive systems. In addition to that, the review goes into topics such as tissue engineering, regenerative medicine, biomedical robotics, automation, biomechanics, and the utilization of green biomaterials. The purpose of this analysis is to provide insights that will enhance continuing research and development efforts in engineering-driven biomedical breakthroughs, ultimately contributing to the improvement of healthcare. These insights will be provided by addressing difficulties and highlighting future prospects.

## Research Highlights

• Integration of engineering into diagnostics leads to early disease detection through medical imaging.
• Biosensors offer cost-effective, simple, and reliable early detection of abnormal health parameters. A smart drug delivery system requires fewer drugs compared to conventional methods.
• Use of natural materials will enhance the biocompatibility of nanomaterials.
• Nanomaterial enhanced tissue regeneration.

## GRAPHICAL ABSTRACT

**Figure:**
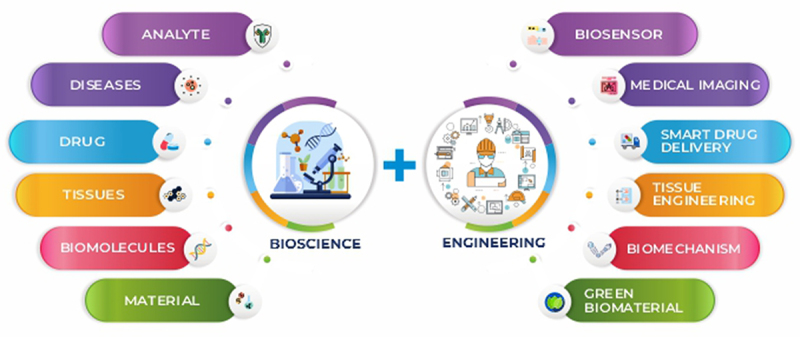

## 1. Introduction

The dynamic nexus between engineering and biomedical sciences has significantly changed the healthcare environment. The area of biomedical engineering, which emerged from the necessary cooperation between engineers and doctors in the middle of the 20th century, is examined in this article for its diverse effects [1]. Biomedical engineering is a unique study that combines elements of biology, physiology, and medicine with engineering disciplines like electrical, mechanical, chemical, and materials engineering [2]. It began as an interdisciplinary specialist within established domains. The goal of this article is to give a thorough review of the revolutionary effects on healthcare that the dynamic synergy between engineering and biomedical sciences has brought about. We explore applications including medical equipment management, biocompatible prosthesis development, and medical device advancement, highlighting the development of biomedical engineering from its interdisciplinary origins to a distinct field. The article endeavors to illuminate the nascent subject of green biomaterials, highlighting the increasing importance of eco-friendly solutions in biological applications, while extending beyond these traditional field [3].

Biomedical engineering makes a substantial technological contribution to healthcare through ongoing technological advancements. Notable advancements in patient care, cost containment, and ease of medical procedures can be found in telemedicine, electronic health records, and AI-driven diagnostics [4]. Our goal is to present a thorough analysis of the most current developments in electronic health records, which have the potential to save patient data more comprehensively, promote better provider-to-provider contact, and ultimately improve patient outcomes [5]. Beyond applications to humans, biomedical engineering acknowledges the universalism of biological principles in all living things. Bioinformatics, biomechanics, biomaterials, biomedical optics, tissue engineering, genetic engineering, neural engineering, pharmaceutical engineering, hospital and medical device engineering, and other subfields within biomedical engineering are all explored in this article [6].

Important subtopics like drug delivery, tissue engineering, regenerative medicine, medical imaging, biomedical sensors, and biomedical robotics and automation, biomechanics and green biomaterials including green nanotechnology are covered in detail in this thorough overview. In addition to highlighting current developments in biomedical engineering, it highlights the ground-breaking influence of engineering on healthcare. This publication, with its emphasis on the broader scope, adds a nuanced viewpoint to the literature and provides insightful information that will be beneficial to scholars, practitioners, and policymakers alike.

## 2. Medical imaging and diagnosis

The process of visually representing the composition and operation of the various human tissues and organs for clinical purposes and scientific research into the normal and abnormal architecture and physiology of the body is known as medical imaging. Medical imaging methods are used to identify anomalies, cure illnesses, and reveal interior structures hidden behind the skin and bones [7]. Healthcare science has evolved from medical imaging. It’s a crucial component of biological imaging and encompasses radiology, which makes use of imaging technologies like thermography, medical photography, electrical source imaging (ESI), digital mammography, tactile imaging, magnetic source imaging (MSI), medical optical imaging, single-photon emission computed tomography (SPECT), endoscopy, MRI, magnetic resonance spectroscopy (MRS), positron emission tomography (PET), and ultrasonic and electrical impedance tomography (EIT) [8].

Among these technologies, some of most advanced and widely used ones are digital mammography, sonography, PET, MRI, CT, and SPECT. These techniques have been developed and improved over the years, and their working principles, applications in medical laboratories, and advancements in imaging techniques can all be used to describe them. Their benefits and uses in the identification, monitoring, and healing of various conditions like trauma, cancer, neurological disorders, and cardiovascular disease helps to clinicians frequently employ these methods because they can quickly and easily manage diseases using photographs [9]. However, these methods also have some limitations and challenges, such as high cost, radiation exposure, low resolution, noise, artifacts [10].

**Figure 1.**
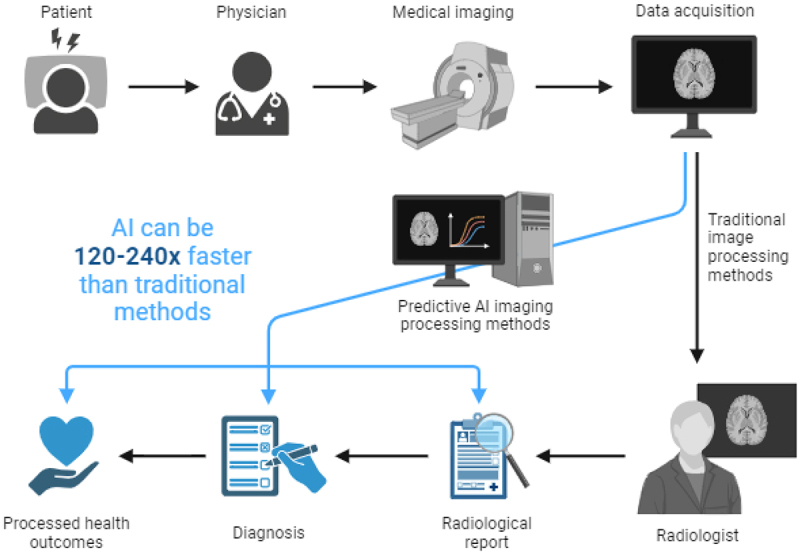
Outlines the diagnostic workflow from patient presentation to CT scan and subsequent steps in the diagnostic process. (Copyright,2023, Biorender).

To overcome these challenges and enhance the performance and accuracy of medical imaging and diagnostics, artificial intelligence (AI) has been integrated into these methods in recent years. AI integration has significantly advanced medical imaging and diagnostics in recent years. Engineer-developed AI algorithms have changed the diagnostic workflow significantly. In addition to the image acquisition and analysis, AI can also significantly enhance the image processing and diagnosis process that follows a CT scan, which is one of the most advanced and widely used medical imaging techniques. AI considerably speeds up the examination of medical images, with processing speeds up to 240 times faster than conventional methods [11]. AI is essential to image processing that follows a CT scan in the diagnosis process shown in Figure 1 By using AI algorithms, the images can be quickly and accurately processed, segmented, classified, and annotated, which can improve the diagnostic workflow’s efficiency and accuracy. AI can also assist the radiologists and clinicians in detecting, diagnosing, and treating various diseases, such as cancer, stroke, brain tumors, etc [12]. Furthermore, AI can provide useful insights and recommendations to the medical practitioners, such as the best treatment options, the prognosis and survival rate of the patients, the potential risks and complications, etc [13]. When radiologists and AI work together, a thorough assessment is guaranteed, which results in quicker and more accurate diagnoses and better patient outcomes overall.

Engineering is essential to the field of medical imaging and diagnosis because it advances image processing methods that improve diagnostic accuracy. Sophisticated algorithms and computational techniques are used in conjunction with healthcare to extract valuable information from medical imaging through synergistic teamwork. These developments enable medical personnel to diagnose patients with more efficiency and precision. Moreover, engineering solutions make it possible to extract complex patterns and characteristics that would be invisible to the unaided eye. In addition to improving image quality, engineering solutions make it possible to extract complex patterns and characteristics that would be invisible to the unaided eye. Combining engineering expertise with medical imaging opens up new avenues for understanding and treating a wide range of medical disorders in addition to improving diagnostic skills [9].

One of the most helpful technologies for medical diagnosis and surgery that benefits from engineering and machine learning is medical image processing. This technology are providing much more valuable information from scans or MRIs by ingesting and classifying 2D, 3D, and even 4D digital photographs composed of an array of pixels with different intensities [14]. The images originate from a range of imaging systems, including micro CT, MRI, CT, and FIB-SEM scanners. By applying morphological and segmentation techniques, the images can be improved and labeled as body parts or regions of interest. A machine learning algorithm that has been taught to identify these regions may then be applied to the resultant data and image. Using their technical knowledge, users can also predefine the imaging’s precise settings and features, such as the shapes, areas, and image pixel histogram of the regions of interest [15]. Typically, a portion of the available data entries for a specific number are utilized for testing, while the remainder are used for training. For training to comprehend the features, a specific machine learning algorithm is chosen. Principal component analysis, support vector machines, convolutional neural networks, and other techniques are a few examples. The trained algorithm is then expected to identify the characteristics and classify the testing image. AI has been used in radiology imaging studies for organ and lesion segmentation, image registration, fiducial/marker detection, radionics, and other purposes. Like radiology, deep learning has replaced traditional AI in this field [11]. Table 1 presents the list of medical devices equipped with AI technology that have been approved by the US FDA for use in cancer radiology-related applications.

**Table 1.** Presents a list of medical devices equipped with AI technology that have been approved by the US FDA for use in cancer radiology-related applications [286].

Serial No.	Year of approval	Imaging Modality	Name of the device	Description about the device and its role
1.	2015	CT	ClearRead CT (Riverain Technologies LLC.)	Providing support for reviewing chest multi-slice computed tomography (CT) scans and identifying potential nodules that require radiologist attention.
2.		Mammography	Transpara (ScreenPoint Medical BV)	Aiding physicians in the interpretation of screening mammograms, aiding in the identification of suspicious areas indicative of breast cancer.
3.	2016	Transrectal Ultrasound (TRUS)	SmartTarget (SmartTarget Ltd.)	Participating in image-guided interventional and diagnostic procedures related to the prostate gland.
4.		CT	LungQ (Thirona Corp.)	Aiding in diagnosing and documenting abnormalities in pulmonary tissue images, specifically extracted from CT thoracic datasets.
5.	2017	Ultrasound	AmCAD-US (AmCad BioMed Corporation)	A software designed to visualize and quantify ultrasound image data along with corresponding backscattered signals.
6.		Mammography	QuantX (Quantitative Insights)	An AI-enhanced diagnostic system designed to assist in achieving accurate diagnoses of breast cancer.
7.		CT	Veye Chest (Aidence BV)	Assistance in the detection of pulmonary nodules from CT scans.
8.	2018	MRI, CT	Arterys Oncology DL (Arterys)	An AI-powered, cloud-based medical imaging software designed to automatically measure and track lesions and nodules in both MRI and CT scans.
9.		Mammogrphy	QVCAD (QView Medical Inc.)	An assistance tool aimed at detecting mammography-occult lesions in areas that were not initially identified as having suspicious findings.
10.		Mammography	HealthMammo (Zebra Medical Vision Inc.)	Processing and analyzing mammograms to identify suspected lesions indicative of breast cancer.
11.		CT, MRI	Arterys Oncology DL (Arterys Inc.)	Assisting in the oncological workflow by aiding users in confirming the presence or absence of lesions. This application supports anatomical datasets such as CT or MRI scans.
12.		Ultrasound	AmCAD-UT (AmCad BioMed Corporation)	Providing support in the analysis of thyroid ultrasound images.
13.		Mammography	Mia -Mammography Intelligent Assessment (KheironMedical Technologies Ltd.)	Offering assistance in the detection of breast cancer through the analysis of mammograms
14.		MRI,CT	Arterys MICA (Arterys)	A platform powered by AI for the analysis of medical images, including MRI and CT scans.
15.		CT, MRI, X ray	SubtlePET (Subtle Medical)	An AI-driven technology that enables medical centers to provide quicker and safer patient scanning experiences, simultaneously improving exam throughput and provider profitability.
16.	2019	Mammography	cmTriage (CureMetrix)	A software utilizing AI for the triage of mammography cases.
17.			Deep Learning Image Reconstruction (GE Medical Systems)	
18.		Mammography	Auto Lung Nodule Detection (Samsung Electronics Co. Ltd. (parent company: Samsung Group)	Breast cancer detection for diagnostic support from mammograms
19.		MRI	JPC-01K (JLK Inspection Inc.)	Offering diagnostic support through the detection of prostate cancer using MRI images.
20.		Mammography	syngo.Breast Care (Siemens Healthcare GmbH (parent company: Siemens AG))	Providing interpretation and reporting services to offer diagnostic support using mammograms.
21.		CT	Aquilion ONE (TSX-305A/6) V8.9 with AiCE (Canon MedicalSystems Corporation)	A device capable of capturing and displaying cross-sectional volumes of the entire body, including the head, with the unique ability to image whole organs within a single rotation.
22.		Digital Breast Tomosynthesis (DBT)	ProFound AI for Digital Breast Tomosynthesis (iCAD Inc.)	A software device for computer-assisted detection and diagnosis (CAD) designed to aid in the interpretation of DBT exams.
23.			RayCare 2.3 (RaySearch Laboratories)	An oncology information system utilized to facilitate workflows, scheduling, and the management of clinical information for oncology care and post-treatment monitoring.
24.		Mammography	Breast-SlimView (Hera-MI SAS)	Providing diagnostic support by detecting breast cancer through the analysis of mammograms.
25.		Mammography	Vara (Merantix Healthcare GmbH)	Assistance in breast cancer screening and triage through the analysis of mammograms.
26.		DBT	ProFound AI Software V2.1 (iCAD)	A CAD software device developed to be used simultaneously by interpreting physicians during the assessment of Digital Breast Tomosynthesis (DBT) images.
27.		Mammography	Transpara (ScreenPoint Medical)	A device designed to assist physicians concurrently while interpreting screening mammograms from compatible FFDM systems. Its purpose is to help identify regions that appear suspicious for breast cancer and evaluate the likelihood of malignancy.
28.		MRI	QyScore software (Qynapse SAS)	Automating the process of labeling, visualizing, and quantifying the volumes of segmentable brain structures and lesions from MRI images.
30.	2020	Mammography	JBD-01K (JLK Inspection Inc.)	Providing diagnostic support through the detection of breast cancer using mammograms.
31.		CT	InferRead CT Lung (Beijing Infervision Technology Co. Ltd.)	A tool designed for lung cancer screening and management through the analysis of CT scans.
32.		Mammography	b-box (b-rayZ GmbH)	Evaluating the quality of mammography images and determining breast density using mammograms.
33.		Mammography	densitasAI (Densitas Inc.)	Offering support for the assessment of breast density using mammograms.
34.		CT	Broncholab (Fluidda Inc)	Aiding in diagnosing and documenting abnormalities in pulmonary tissue images obtained from CT thoracic datasets.
35.		CT	Syngo.CT Lung CAD (Siemens Medical Solutions Inc. (parent company: Siemens AG))	Aiding in the detection of solid pulmonary nodules while reviewing multi-detector computed tomography (CT) exams of the chest.
36.		DBT	Genius AI Detection (Hologic, Inc.)	A software device designed to detect potential abnormalities in breast tomosynthesis images.
37.		Mammography	MammoScreen (Therapixel SA)	Assisting in the identification of findings on screening Full Field Digital Mammography (FFDM) acquired with compatible mammography systems and evaluating the level of suspicion associated with them.
38.		Mammography	Visage Breast Density (Visage Imaging)	The software application is designed to be utilized alongside compatible full-field digital mammography systems, supporting radiologists in evaluating breast tissue composition.
39.		Ultrasound	Imagio Breast Imaging System (Seno Medical Instruments, Inc.)	Enables an enhanced classification of breast masses in comparison to using ultrasound alone, incorporating AI-based software.

In fact, one important method in the field of medical imaging and diagnosis is Photoacoustic imaging (PAI). By fusing the benefits of optical and ultrasonic imaging, PAI provides a high-resolution, noninvasive imaging technique [16]. Short laser pulses are employed in photoacoustic imaging to illuminate tissues, which produce photoacoustic waves. Ultrasound transducers then pick up on these waves, enabling the production of fine-grained pictures that provide details about the optical absorption properties of various tissues. With superior contrast and spatial resolution, PAI can be utilized to see things including organs, tumors, and blood arteries [17]. One of the emerging applications of PAI is photoacoustic tomography (PAT), which is a hybrid approach that relies on the acoustic detection of optical absorption from exogenous contrast agents such organic dyes and nanoparticles, or endogenous chromophores like oxy- and deoxy-hemoglobin. In both the optical ballistic and diffusive regimes, PAT produces high-resolution images because ultrasonic scatters far less than light in tissue. Here we have discussed molecular PAT [18].

**Figure 2.**
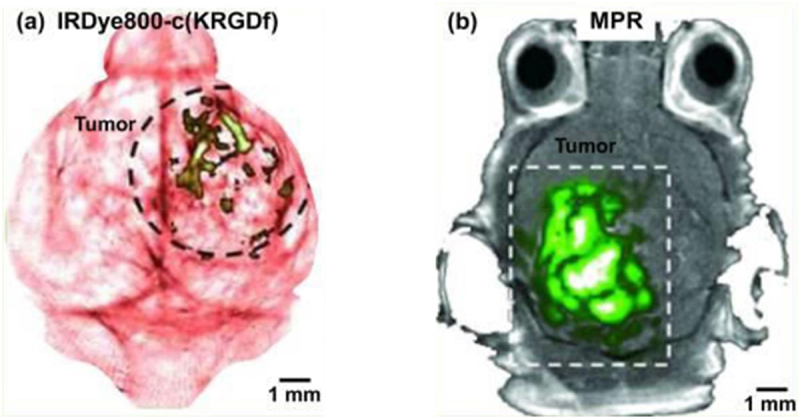
Photoacoustic molecular imaging. (a) PACT of a glioblastoma in a mouse brain enhanced by IRDye800-c(krgdf), which targeted overexpressed integrin αvβ3 in tumor cells. (b) PAT of a glioblastoma in a mouse brain enhanced by tri-modality MRI-PA-Raman (MPR) nanoparticles (adapted with permission from ref [18]. Copyright,2014, NIH public access).

Endogenous PA contrasts are widely distributed and harmless. Examples of these include hemoglobin in red blood cells, melanin in melanoma cells, DNA/RNA in cell nuclei, water in brain edema, and lipids in myelin. They might not, however, have the necessary specificity to monitor biological processes or diagnose illnesses. Molecular PAT allows the visualization of particular cellular functions and molecular processes with the use of exogenous contrasts. In the past several years, a lot of effort has been put into enhancing PAT’s molecular imaging capabilities [19]. Notable progress has been made in raising the detection sensitivity of PAT imaging systems and in developing contrast agents that provide superior contrast enhancement [20]. PAT has demonstrated the ability to do high-sensitivity molecular imaging through the utilization of several exogenous contrast agents, such as fluorescent proteins, chemical dyes, nanoparticles, micro bubbles, and reporter gene products [21,22]. For instance, targeting overexpressed integrin αvβ3 in brain glioblastoma with IRDye800-c (KRGDf) was one of the earliest examples of molecular PAT (Figure 2a). According to reports, the claimed PA detection sensitivity for exogenous contrast agents ranges from millimolar to picomolar due to their radically differing optical absorption qualities [23].

Nanoparticles can be readily tailored for PA molecular imaging by adjusting their size, shape, and composition to get the ideal peak absorption wavelength, in contrast to organic dyes. For PA molecular imaging, a variety of nanoparticles have been employed, particularly for sentinel lymph node mapping and cancer detection. For instance, Kircher et al. have developed a triplemodality MRI-photoacoustic-Raman nanoparticle technique for molecular imaging of brain tumors (Figure 2b) [24]. An incident laser energy of only 8 mJ/cm2 was sufficient to obtain a high detection sensitivity of 50 pM.

Medical image processing is thought to be crucial for building precise and customized patient simulations and intricate models that may be used to assess and forecast surgical results. Through the utilization of medical photos, scientists and professionals can improve the accuracy and efficiency of virtual models, allowing for more realistic representation of anatomical variances in real life and more informed surgical decision-making [25]. Pedicle screw fixation is a frequent treatment for thoracolumbar fractures and is widely used to treat diseases or injuries of the spine. This operation may result in post-operative problems that cause back pain or require revisions. When using both actual and simplified screw designs, finite element (FE) models should be verified before being utilized to forecast surgical results. Human vertebrae with two pedicle screws that are patient-specific CT-based FE models. These models are validated, and the screws’ size and geometry are used to assess how the screws’ mechanical characteristics affect the screw-vertebra structure. Even though pedicle screws are widely used in modern clinical practice, mechanical problems such as screw breaking and loosening are common in spinal fixation and might result in revision surgery in approximately 6% of cases. To improve the results of this operation, parameters connected to the procedure should be optimized. While surgeons use anatomical measurements from CT scans to determine the ideal screw size, insertion point, and orientation, FE models are effective tools for mechanically evaluating the stability of various instrumented spine configurations under various loading scenarios [26].

## 3. Advancement in biosensors

Sensors are devices that have the ability to sense, or respond to, a given parameter and, in accordance with a particular law, convert it into an output signal that is useful [27]. Usually, a transducer element that directly reacts to the parameter being monitored and generates a signal output that may be used is combined with associated electronics to form the sensor. Biological sensors are unique electronic devices that have the ability to convert biological information into readily measured electric signals, out of all types of sensors [28]. Physical, chemical, and biological quantity sensors are the three categories into which biomedical sensors can be classified based on the kind of human physiological data they are intended to identify. Biomedical sensors are widely used in laboratory analytical applications, clinical and portable diagnostics, and medical image processing and diagnostic [29]. Use of these biosensor will provide a cost-effective solution for remote health monitoring, which is further divide in to wearable and non- wearable sensors. Additionally, these devices will enable medical staff to evaluate patients’ health problems, monitor vital physiological indications in real-time, and receive input from locations that are far away [30].

In recent year use of nanotechnology specially introductions of nanoparticles in biosensor has increased its limit of detections and produced higher-quality results. Nanoparticles have special properties with respect to bulk material they provide high surface to volume ratio, along with high magnetic, optical and electrical field. Table 2 presents the list of nanomaterials used for biosensing. The utilization of metallic nanoparticles in biosensors has become widespread, encompassing a diverse range including gold, silver, platinum, iron, titanium, and copper nanoparticles [60]. Additionally, nonmetals and metalloids like silicon, phosphorous, boron, and carbon contribute to the synthesis of nanomaterials, either independently or in combination with metals to form composites [30]. Another class of materials, biopolymers, plays a pivotal role in constructing Nano biosensors, primarily involved in surface modification and coating [61]. While various nanoparticles find application in biosensors, gold nanoparticles (GNPs) stand out due to their unique surface functions and qualities. One distinctive property of GNPs is surface plasmon resonance (SPR), a phenomenon where local electron confinement changes based on particle size and shape, reflected in the absorption maximum and color of the colloidal solution [62]. This feature makes GNPs particularly valuable in biosensors. The interaction between analytes and GNPs alters the characteristics of the nanoparticles, such as conductivity, leading to detectable signals in diagnostics. SPR, along with redox behavior, contributes to the effectiveness of GNPs in biosensing [63]. The widespread use of GNPs in biosensor production is evident in the application of various gold nanomaterial shapes, including nanospheres, nanorods, and nanopatterns [64]. For instance, a biosensor utilizing gold nanorods (GNRs) was developed for Hepatitis B virus detection. Through an adsorption method, the gold nanorods (AuNRs) underwent modification with the identified virus antigen, resulting in a red shift in the longitudinal band [65]. Similar success stories are reported in disease diagnostics, such as the application of gold dot nanopatterns on indium tin oxide for HIV-1 detection and spherical GNPs in an optical biosensor for PSA (prostate-specific antigen) detection. Notably, in environmental science, GNPs have been instrumental in creating electrochemical biosensors for detecting pesticides, including organophosphorus compounds. These sensors exhibit an impressive molar range for detection limits, reaching as low as fM and pM [64]. This versatility and sensitivity underscore the significant role of GNPs in advancing biosensor technology across various fields. Gold nanoparticles (AuNPs) contribute to biosensing by facilitating the detection of various antibiotics through calorimetry, including amoxicillin, chloramphenicol, kanamycin, tetracycline, oxytetracycline, and streptomycin [66]. Additionally, significant strides have been made in the detection of hazards to human health using silicon and magnetic nanoparticles. Magnetic nanoparticles (MNPs) are employed in sensing applications either by attaching them directly to transducer systems or by dispersing them across the detecting sample and then attracting an external magnetic field. Silicon nanoparticles (SiNPs) play a crucial role in detecting antibiotics like penicillin, tetracycline, and oxytetracycline, while MNPs excel in detecting biomarkers of cancer cells, CEA, Escherichia coli, and Staphylococcus aureus. The strong physiological and electrochemical affinity of these nanomaterials for specific compounds holds promise for achieving desired health conditions [67].

**Table 2.** Presents the list of nanomaterials used for biosensing.

S.No	Nanomaterial	Synthesis Method	Morphology	Application	Limit of Detection	References
1	Au/CdS QDs/TNTs	Electrochemical	–	Detection of cholesterol and hydrogen peroxide (H_2_O_2_)	0.012 µM 0.06 µM	[31]
2	Au NP-MoS_2_-rGO	Hummer’s method	Flower shape	Detection of Carcinoembryonic antigen (CEA)	0.084 ng mL^−1^	[32]
3	Au/rGO	Electrochemical		Detection of miENA-122 Biomarker	1.73 pM	[33]
4	Ag NPs	Colorimetric	Spherical	Determination of H_2_O_2_ Glucose Fe^2+^	0.032 µm 0.29 µm 0.54 µm	[34]
5	Ag/Pd NPs	Electrochemical	Wrinkled, paperlike structure	Detection of Ractopamine Clenbuterol Salbutamol	1.52 pg mL^−1^ 1.44 pg mL^−1^ 1.38 pg mL^−1^	[35]
6	Ag@CQDs-rGO	Electrochemical	Spherical	Detection of Dopamine	0.59 nm	[36]
7	Ag NP-MWNT	Electrochemical		Detection of Glucose	0.01 mM	[37]
8	Pt NPs	Voltammetric	Irregular shape	Detection of Adrenaline	2.93 × 10^−4^ mol L^−1^	[38]
9	Pt-Fe_3_O_4_@C	Amperometric	Spherical	Detection of Sarcosine	0.43 µm	[39]
10	Pt@CeO_2_ NM	Electrochemical	Sphere	Detection of Dopamine	0.71 nM	[40]
11	Cu/rGO-BP	Electrochemical		Detection of Glucose	11 µm	[41]
12	Ni/Cu MOF	FET(field-effect transistor)	Crystal	Detection of Glucose	0.51 µM	[42]
13	NiO/PANINS	Amperometric	–	Detection of Glucose	0.06 µM	[43]
14	Co_3_O_4_-Au	Photo-electricalchemical	–	Detection of miRNA-141	0.2 pM	[44]
15	ZnO-rGO	Cyclic Voltammetric	–	Detection of Dopamine	8.75 ± 0.64 pM	[45]
16	ZnO NFs	Optical	flower petals Shape	Detection of Amyloid	2.76 µg	[46]
17	Cr doped SnO_2_ NPs	Voltammetric	Spherical	Detection of Riboflavin	107 nM	[47]
18	TiO_2_/APTES	Impedimetric	Irregular	Detection of Glucose	24 µmol	[48]
19	MoO_3_@RGO	Electrochemical	Globular	Detection of Breast cancer	0.001 ng mL^−1^	[49]
20	Graphene QDs	Fluorescence	Round	Detection of Lung cancer^+^	0.09 pg mL^−1^	[50]
21	NSET amptamer@Fe_3_O_4_@GOD and MoS_2_	Magnetic fluorescence	–	Detection of Tumor cell(EpCAM)	1.19 nM	[51]
22	Au NPs@PDA@CuInZnS QDs	Electrochemiluminescenece	Round	Detection of P53 gene	0.03 nmol L^−1^	[52]
23	Si NWs	FET	–	Detection of Dengue virus	2.0 fM	[53]
24	G/Au NR/PT	Electrochemical	–	Detection of HPV DNA	4.03 × 10^−14^ m L^−1^	[54]
25	Co_3_O_4_-CNT/TiO_2_	Photoelectrochemical	–	Detection of Glucose	0.16 µM	[55]
26	CNT thin-film transistor (TFT)	Thin film transistor (TFT)	–	Detection of DNA	0.88 µg L^−1^	[56]
27	GQDs-MWCNTs	Electrochemical	–	Detection of Dopamine	0.87 nM	[57]
28	PAMAM dendrimer	Optical fiber	Cylindrical Structure	Detection of Dengue E Protein	19.53 nm nM^−1^	[58]
29	SAM/NH_2_rGO/PAMAM	SPR	Globular	Detection of Dengue E protein	0.08 pM	[59]

Silver nanoparticles (AgNPs) have also emerged as highly desirable entities in the realm of biosensing, owing to their remarkable qualities such as a vast surface area, favorable electrocatalytic capacity, and exceptional optical properties [68]. The field of biosensor research and modification has extensively utilized silver nanoparticles, with applications ranging from glucose sensors to various nanobiosensors. For instance, silver nanoparticles have been employed in the creation of glucose sensors and have taken diverse forms in nanobiosensors [69]. Meanwhile, carbon, in the form of nanotubes and sheets, has found utility in protein, glucose, DNA, lateral flow, and impedance biosensors. However, it is essential to note that excessive consumption of carbon nanomaterials may lead to adverse effects such as fibrosis and inflammation.The processes of biosensors are intricately linked to the analyte to be examined and its specific application. Techniques such as ELISA and other immunoassay methods are commonly employed for protein detection, while the electrochemical and optical properties of carbon nanostructures allow for the detection of a wide range of analytes [70]. The rapid advancement in silicon technology has paved the way for the development of nanostructures or nanohybrids, contributing to the production of high-quality sensors for biosensing applications [67].

Recent advancements have also ushered in new applications of polymers in sensor technology. These polymers, characterized by good mechanical and physicochemical properties, have gained attention due to their specificity, nontoxicity, renewability, and biodegradability [71]. They find utility in the detection of enzymes, proteins, infections, and extracellular products, particularly in point-of-care devices, medical diagnostics, and cellular imaging [72]. Biopolymers like starch, collagen, cellulose nanofibers, and peptides such as glycine and leucine are among the frequently mentioned in this context. A summary of the most important biosensor parameters is given in Table 2, which also includes the kind of nanomaterial used, the synthesis techniques used, the morphological properties, the intended applications, and the limit of detection attained. When assessing the wide range of biosensor technologies and their possible applications in healthcare monitoring and diagnostics, this table is a useful resource.

### 3.1. Wearable biosensors

**Figure 3.**
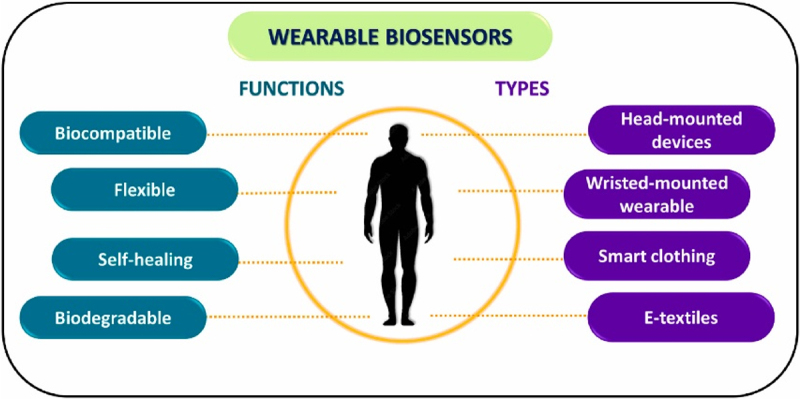
Diagrammatic representation of the functions and the types of wearable biosensors for healthcare and biomedical applications.

Wearable sensor, defined by its features such as mobility, wearability, sustainability, simple operation, and interactivity, represents a transformative integration of computing into the physical world worn on the human body [73]. Comprising sensors, discs, displays, and computer components, wearable devices create a digital environment that enhances comfort and convenience through wireless network connections [74]. Existing wearable medical devices, including watches, bracelets, armbands, ‘smart’ eyewear, and more, serve various purposes in healthcare, consumption, and industrial and military applications.The sensor, a crucial component of many modern wearables, plays a role similar to human skin by sensing changes in the external environment and responding appropriately. Examples of wearable sensors include fitness trackers, smart clothing, smartwatches, and continuous glucose monitors (CGM) [75]. The primary application areas for wearable devices include the medical domain (blood pressure, heart rate, and blood sugar monitoring), healthcare domain (exercise monitoring), consumption domain (infotainment), and industrial and military domains (data collection and display) [76]. Figure 3 shows the functions and types of wearable biosensors for healthcare and biomedical applications.

The future of wearable technology is poised for developments integrating micro electromechanical system (MEMS) devices, sensor fusion, and the use of different data obtained by sensors connected to the Internet [77]. MEMS sensors, particularly in sports health data, play a significant role in wearable sensor applications. Flexible sensors, a novel type of sensor relying on flexible circuitry and materials, are gaining importance in wearable medical devices due to their human adaptability [78]. These sensors are thin, soft, and elastic, making them suitable for direct placement on the human body. The signal conversion mechanisms of flexible wearable electronic sensors include piezoresistive, capacitive, and piezoelectric components [79]. To advance further, flexible wearable electronic sensors must make strides in material synthesis, fabrication techniques, and device integration. Scientific progress in multifunctional integration, complex environmental analysis, and new sensing principles is crucial [80]. Wearable sensors also hold expansive application opportunities, particularly in the realms of nanomaterials and nanotechnology research [81].

**Figure 4.**
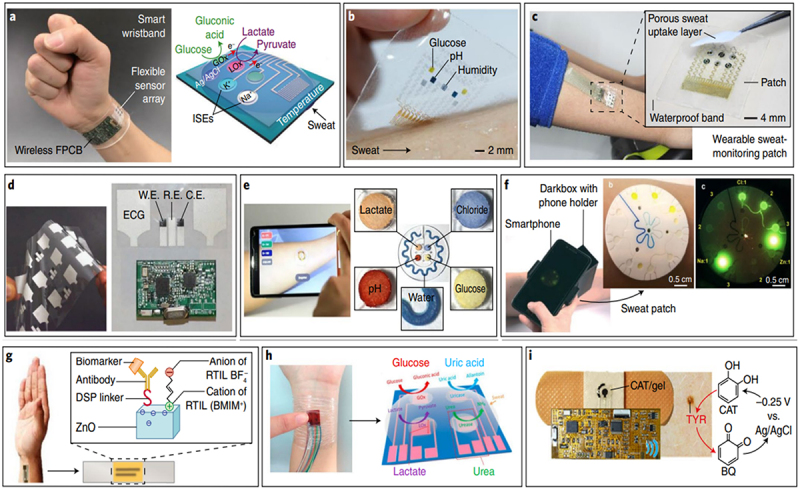
(a) Depiction of integrated wearable sensor arrays for multiplexed perspiration analysis applied to wrist with schematic representation of sensing array configuration. (b) depiction of graphene-based sweat sensor array for diabetes monitoring applied to human forearm. (c) depiction of wearable sweat monitoring patch for sweat-based glucose monitoring and therapy applied to human forearm during exercise.(d) depiction of wearable chemical-electrophysiological hybrid biosensor configuration for real-time health and fitness monitoring with example of screenprinted electrodes. (e) depiction of colorimetric microfluidic sweat sampling device configuration for chemical analysis of sweat with representation of sweat-filled device and smartphone-based signal analysis. (f) depiction of fluorometric skin-interfaced microfluidic platform for the measurement of chloride, sodium and zinc in exercise induced sweat. (g) schematic representation of wearable diagnostic antibody-based biosensor targeting detection of IL-6 and cortisol in human sweat using room temperature ionic liquids for enhanced antibody operational stability. (h) schematic representation of self-powered multifunctional electronic skin used for continuous monitoring of lactate, glucose, uric acid, and urea in exercise-induced sweat using piezoelectric-linked enzymatic biosensors. (i) depiction of wearable tyrosinase sensing bandage for noninvasive melanoma screening. With permission, this image has been reproduced [288]. Copyright 2021 nature biotechnology.

Figure 4a figure illustrates integrated wearable sensor arrays for multiplexed perspiration analysis, applied to the wrist. The schematic representation Figure 4b depicts the configuration of the sensing array. Depicting a graphene-based sweat sensor array for diabetes monitoring on the human forearm, this figure showcases the sensors’ arrangement and their role in monitoring glucose levels. Figure 4c features a wearable sweat monitoring patch for sweat-based glucose monitoring and therapy, applied to the human forearm during exercise, demonstrating its functionality and application. Figure 4d showing a wearable chemical-electrophysiological hybrid biosensor configuration for real-time health and fitness monitoring, this figure includes an example of screen-printed electrodes. This Figure 4e presents a colorimetric microfluidic sweat sampling device configuration for chemical analysis of sweat, including a representation of the sweat-filled device and smartphone-based signal analysis. Figure 4f illustrates a fluorometric skin-interfaced microfluidic platform for measuring chloride, sodium, and zinc in exercise-induced sweat, this figure highlights the microfluidic setup and its use in analyzing sweat composition. This Figure 4g provides a schematic representation of a wearable diagnostic antibody-based biosensor targeting IL-6 and cortisol detection in human sweat, utilizing room temperature ionic liquids for enhanced stability and Figure 4h Depicting a self-powered multifunctional electronic skin for continuous monitoring of lactate, glucose, uric acid, and urea in exercise-induced sweat, this figure showcases piezoelectric-linked enzymatic biosensors.

Overcoming challenges in achieving noninvasive measurements is a significant focus, given that the body contains all the biosensors’ measurement objectives. Wearable, highly sensitive biosensors can alleviate psychological and physiological burdens associated with traditional blood collection methods. Google and Novartis, for instance, are developing a contact lens sensor equipped with a miniature electrochemical sensor in a hydrogel matrix to measure glucose levels in tears [82]. As technology continues to advance, wearable technology is poised to play an increasingly pivotal role in healthcare and various other domains.

Table 3 serves as a comprehensive guide, delineating essential attributes of various wearable devices. This table encapsulates key parameters such as the name of the device, body fluid targeted for analysis, underlying working principle, intended applications, and specificity or limit of detection achieved.

**Table 3.** Present the list of various wearable devices.

S. No	Name of the device	Body Fluid	Working Principle	Application	Specificity or Limit of Detection	References
1	Mouthguard	Saliva	Electrochemical	Analysis of Uric acid	0–1 mM	[83]
2	Wristband and headband	Sweat	Electrochemical	Analysis of Glucose, sodium, and lactate	Glucose: 0–200 μM Na+:10–160 mMLactate: 30 mM	[84]
3	Contact lens	Tear	Optical	Analysis of Glucose	0–50 mM	[85]
4	Wearable patch	Sweat	Electrochemical	Analysis of Lactate	3–100 mM	[86]
5	Fex Coy O4 -rGO Flexible electrode	Tear	Electrochemical	Analysis of Glucose	0.07 μM	[87]
6	Wristband	Sweat	Electrochemical	Analysis of Cytokines (interleukin-6, interleukin-8, interleukin-10, and tumor necrosis factor-a)	-	[88]
7	Inverse opal carbon rod electrode	Tear	Electrochemical	Analysis of Lactoferrin and Glucose	Lactoferrin: 0.1–5 mg/mL	[89]
8	Graphene-based platform	Intestinal fluid (ISF)	Electrochemical	Analysis of Glucose	0.006–0.7 mM	[90]
9	Mouth guard	Saliva	Electrochemical	Analysis of Glucose	1.75 –10,000 μM	[91]
10	Wearable patch	Sweat	LSPR	Analysis of Cortisol	0.1–1000 nM	[92]
11	Contact lens	Tear	Optical	Analysis of Glucose	23 μM to 1.0 mM	[93]
12	Conductive cotton fibers	ISF	Electrochemical	Analysis of Lithium	6.3 × 10–5 to 6.3 × 10–2 2 M	[94]
13	Pacifier	Saliva	Electrochemical	Analysis of Glucose	0.1–1.4 mM	[95]
14	A high-density polymeric Micro Needle (MN) array-based (PMNA) sensing patch	ISF	Electrochemical	Analysis of pH	-	[96]
15	Microelectrodes	ISF	Electrochemical	Analysis of Glucose	0–32 mM	[97]
16	The eye patch	Tear	Optical	Analysis of pH, protein, ascorbic acid, and glucose	Protein: 0.17 g/L, Glucose: 7.0 μM Ascorbic acid: 3.0 μM	[98]
17	A silk- MN patch	ISF	Electrochemical	Analysis of Glucose	3–18 mM	[99]
18	Wearable patch	Sweat	Electrochemical	Analysis of Glucose	1.25–850 µM	[100]
19	MN	ISF	Optical	Analysis of microRNA	miRNA-141: 14 pM miRNA-155: 6 pM	[101]
20	Contact lens	Tear	Optical	Analysis of pH, glucose, protein, and nitrate ions	0.25 pH units Glucose: 1.84 mM Protein: 0.63 g Nitrate ions: 24.43 μmol	[102]
21	WO3/Au/WO3 electrode	Tear	Field-effect transistor (FET)	Analysis of L-cysteine	0.043 × 10–6 M	[103]
22	Skin patch	Sweat	Electrochemical	Analysis of Glucose, pH	Glucose: 0.2–1 mM pH: 4–6	[104]
23	Eyeglasses	Tear	Electrochemical	Analysis of Creatinine	1.6–2400 μM	[105]
24	Superwettable band	Sweat	Optical	Analysis of Cl, pH, glucose, and calcium	pH: 6.5–7.0 Cl: 100 mM	[106]
25	MN array	ISF	Electrochemical	Analysis of Glucose, lactate, and alcohol	Glucose : 0–40 mM Lactate : 0–28 mM Alcohol: 0–100 mM	[107]
26	Wristband	Saliva	Electrochemical	Analysis of Phenylalanine	20–100 mM	[108]
27	Headband	Sweat	Electrochemical	Analysis of Sodium	0.21–24.54 mmol/L	[109]
28	Contact lens	Tear	Electrochemical	Analysis of Glucose	0.4 mM	[110]
29	MN	ISF	Electrochemical	Analysis of Lactate and glucose	0.1–10 mM	[111]
30	MN	ISF	Electrochemical	Analysis of Apomorphine	0.6–0.75 μM	[112]
31	Wearable patch	Sweat	Electrochemical	Analysis of Chloride	10,000–150,000 µM	[113]
32	MN	ISF	Electrochemical	Analysis of Alcohol	0–80 mM	[114]
33	Contact lens	Tear	Electrochemical	Analysis of Glucose	0.1–0.6 mM	[115]
34	Tattoo	ISF	Acoustic and electrochemical	Analysis of Blood pressure, heart rate, glucose, lactate, caffeine, and alcohol		[116]
35	pH indicator	Saliva	Electrochemical	Analysis of pH (H+)	4–9	[117]
36	MN	ISF	Electrochemical	Analysis of Urea	2.8 µM	[118]
37	MN	ISF	Electrochemical	Analysis of β-hydroxybutyrate	Lac: 1–10 mM HB: 0.0–1.0 mM Glucose:1–10 mM	[119]
38	Glove	Sweat	Electrochemical	Analysis of Alcohol and vitamin C	0–300 μM (for vitC)	[120]
39	Tattoo	ISF	Electrochemical	Analysis of Glucose (in ISF)	0.06 μM	[121]
40	Flexible adhesive patch	Sweat	Electrochemical	Analysis of Urea	5–200 mM	[122]
41	Eyeglasses	Tear	Electrochemical	Analysis of Glucose, alcohol, and vitamins (B2, B6, and C)	B2: 300 μM B6: 500 Μm C: 1000 μM	[123]
42	Mouth guard	Saliva	Electrochemical	Analysis of Lactate	0.1–1.0 mM	[124]
43	MN	ISF	SERS	Analysis of H2 O2	1 μM	[125]
44	Skin patch	Sweat	Optical	Analysis of pH	4–9	[126]
45	wearable fabric	Sweat	Electrochemical	Analysis of Na+ and K+		[127]
46	MN	ISF	SERS	Analysis of Pyocyanin	30 μM	[128]
47	Skin patch	Sweat	Optical	Analysis of Glucose	0–15 mM	[129]
48	Contact lens	Tear	Optical	Analysis of Glucose	0.1 mM	[130]
49	Tattoo	ISF and sweat	Electrochemical	Analysis of Glucose (in ISF)	Glucose: 0–160 µM	[131]
50	Flexible adhesive patch	Sweat	Electrochemical	Analysis of Glucose, lactate, Na+, and K+	Glucose: 0–300 μM Laktate: 5–25 mM, Na +: 5–160 mM K + : 1–32 mM	[132]

### 3.2. Non-wearable biosensors

The field of non-wearable biosensors has brought about a revolutionary change in the healthcare industry by serving as a key component in the integration of engineering and biological sciences [132,133]. These gadgets have evolved into necessary tools, providing quick and easy ways to keep an eye on important health indicators. Blood glucose meters, such as those made by industry titans OneTouch and Accu-Chek, enable diabetics to take measures often and on schedule, improving their ability to control blood glucose levels. Blood pressure monitors from Omron and Welch Allyn have revolutionized the field of cardiovascular health by allowing people to monitor their blood pressure at home, which has led to earlier identification and treatment of hypertension [134,135]. The technological wonders known as pulse oximeters, created by businesses such as Nonin and Contec, offer noninvasive information on pulse rates and oxygen saturation levels, which greatly aids in the evaluation of respiratory and circulatory functions [136].

Digital thermometers, which come in a variety of sophisticated designs from Braun and iProven, provide temperature readings that are both quick and accurate [137]. These thermometers are vital instruments for monitoring fever and a variety of other health concerns. Beyond the realm of individual health, breathalyzers, which were developed by BACtrack and AlcoHAWK, have emerged as essential instruments in the promotion of responsible alcohol consumption. These breathalyzers use breath analysis to determine the amount of alcohol that is present in the blood. Even though they are still in the development phase, Lab on a Chip biosensors provide a glimpse into a future in which miniaturized devices will integrate complicated laboratory operations in a seamless manner [138]. This holds the promise of bringing about innovations that will revolutionize diagnostics and personalized treatment. Essentially, the environment of non-wearable biosensors is a testament to the revolutionary synergy that exists between engineering and biomedical sciences. This synergy is defining a healthcare landscape that is more accessible, proactive, and personalized [139].

### 3.3. Biochip

A highly interdisciplinary field of microanalysis, biochip technology combines computer science, physics, chemistry, molecular biology, microelectronics, and micromechanics [140]. Three processes are involved in the use and preparation of biochips: bimolecular reaction, signal detection and analysis, and biochip preparation [141]. First, using photoconductive in situ synthesis or the microdot method, a vast array of biological macromolecules, including polypeptide molecules, nucleic acid fragments, tissue slices, cells, and other biological samples, were carefully solidified on the support’s surface to create a dense two-dimensional molecular array. The labeled target molecule probe was then used to react with the biological samples that had been processed. Ultimately, specialized instruments scan, detect, and efficiently gather the strength of hybridization signals in a timely manner. A computer then establishes a biological model to enable sample detection and analysis.Numerous biochip classification techniques exist, which are further classified into chip categories such as gene, protein, cell, tissue, and organ-like [29].

#### 3.3.1. Gene chips

Gene chips, sometimes referred to as DNA chips, work on the basis of creating a DNA oligonucleotide probe array in order to identify alterations in sample genes based on the complementary pairing principle between the probe and the target gene’s nucleic acid molecular bases [142]. With the use of precision machinery, optoelectronics, microelectronics, and information technologies, DNA molecules are placed on the support’s surface to create a microarray. Dots of various hues and intensities are created when the DNA molecules on the microarray hybridize with the various fluorescent dye-labeled sample molecules [143]. It may obtain information about hundreds of thousands of genes at a time through the collection and processing of high-throughput image data. SNP, miRNA-microarray, gene expression profiling, DNA methylation, and other chips are already available. Depending on whether oligonucleotide synthesis is required beforehand, there are two types of gene chip preparation techniques [144].

**Figure 5.**
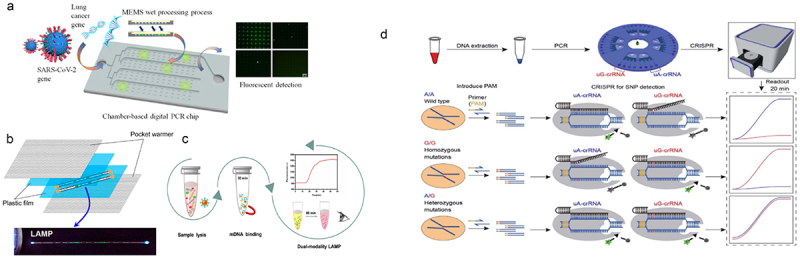
Diagram of integrated microfluidic chips with nucleic acid amplification. (a) dPCR chip for high-throughput, high-sensitivity quantitative measurements of SARS-CoV-2 viral genes. (b) microcapillary LAMP for the detection of nucleic acids. (c) Dual-mode LAMP incorporating magnetic bead separation to determine the methylated Septin9 gene in colorectal cancer. (d) (A CRISPR/Cas12a-based SNP detection genotyping method based on the centrifugal microfluidic device (adapted with permission from ref [148], copyright, 2023 Elsevier).

At the vanguard of genomics research are gene chips, sometimes called DNA microarrays, which are revolutionary instruments that allow for the simultaneous investigation of thousands of genes in a biological sample. These microarrays are made up of tiny DNA patches placed on a solid surface that correspond to particular genes or sequences [145]. Gene expression profiling, which enables researchers to thoroughly analyze gene activity and comprehend how expression patterns vary under various circumstances, is one of the main uses of gene chips. Gene chips are essential for disease research because they provide light on the intricate molecular mechanisms behind different ailments. Scholars can find important genes linked to diseases and possible targets for therapeutic interventions by comparing the gene expression profiles of healthy and sick tissues [143]. Gene chips help identify the genes that are unregulated or downregulated in cancer cells, which aids in understanding the disease’s molecular cause and makes the hunt for diagnostic biomarkers easier [146]. Another crucial use of gene chips is pharmacogenomics, which opens the door to personalized. Medicine by analyzing how different genetic variants affect how each patient reacts to medication. Gene chips help clarify the roles of genes in biological processes through functional genomics research, which goes beyond applications focused on diseases. Furthermore, their potential for use in diagnostic applications offers hope for the creation of assays that make use of distinctive gene expression profiles for the early diagnosis of disease. Gene chips, in summary, contribute to our understanding of genomics in a variety of ways by providing information on gene function, regulation, and the consequences of these findings for both health and sickness [147]. Figure 5 illustrate the integrated microfluidic chips with nucleic acid amplification.

#### 3.3.2. Protein chips

Protein chips are made of microarrays of antigens or antibodies that have been fixed to various support media types [149]. The location and make-up of the fixed molecules in the array are known, and the labeled antigens or antibodies can react with the probes on the chip to be picked up by a particular scanning device. The protein chip points, like a gene chip, a protein to the surface of a solid-phase substance, where it subsequently ‘hybridises’ with the target tissue or cell. Automated instrumentation is then used to analyze the outcome. Similar to their gene-focused counterparts, protein chips are a state-of-the-art proteomics tool. These microarrays allow for the simultaneous study of several proteins in a biological sample since they have immobilized proteins on a solid surface. Protein chips are primarily used to study post-translational changes, quantify protein expression levels, and clarify protein interactions [150]. By using protein chips, scientists can thoroughly examine various biological processes and gain insight into the complex webs of protein-protein interactions that underpin cellular functioning. Protein chips aid in the identification of disease-specific biomarkers and the comprehension of abnormal patterns of protein expression linked to different illnesses, providing information useful for targeted therapies and diagnostics. Furthermore, by identifying possible therapeutic targets and assessing how potential medications affect protein interactions, protein chips are essential to the drug discovery process. Protein chips are essential tools for deciphering the complexity of the proteome and expanding our knowledge of the roles proteins play in health, sickness, and medication responses because of their high-throughput capabilities and adaptability [151].

#### 3.3.3. Cell chips

Within the biochip domain, the terms ‘cell chips’ primarily denote microarray and microfluidic-based cell chips as shown in 5. The microarray cell chip employs cells as a fixed biomolecule in the array and is based on the fundamental concepts of gene and protein chips. It is mostly utilized for high-throughput drug screening, the expression of various genes in cells, etc. The primary purpose of the microfluidic-based cell chip is to assess the intracellular components, metabolic activities, and electrophysiological characteristics of the cells by combining sensing detecting technology with micromachining technology. The advantages of the microarray cell chip over classic cell detection studies are evident in high-throughput, high-performance analysis, whereas the microfluidic cell chip can improve detection efficiency by enabling simultaneous measurement of several cell characteristics [152].

**Figure 6.**
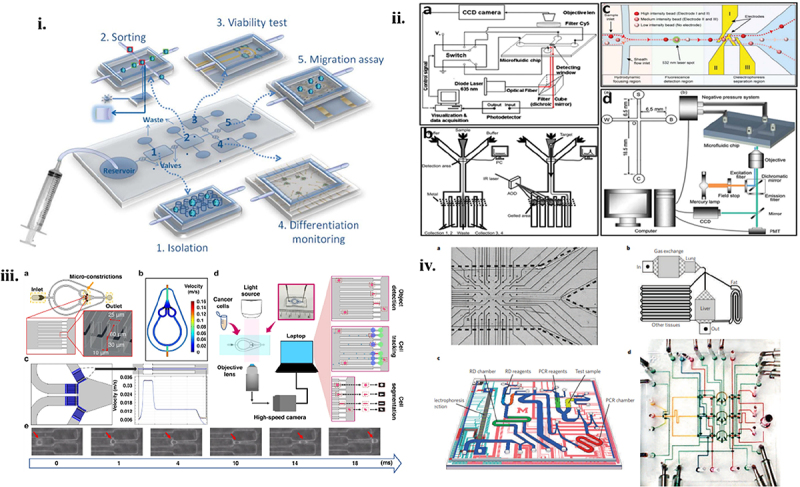
I. Schematic diagram of the microfluidic device. ii. Top view of laminar flow velocity simulation of the microfluidic chip under a 50 μL/min flow rate. With permission, this image has been reproduced [156]. Copyright 2013,Lab on a chip. iii. The fluid flow velocity across the 36 micro constrictions under a 50 μL/min flow rate. IV. Schematic view of the cDC platform’s experimental setup and the computational framework for automatic training set generation, multiple object tracking, segmentation, and cellular deformability quantification (ATMQcD). V. Time-lapse imaging demonstrated the cell deformation and movement process while passing through a micro constriction. With permission, this image has been reproduced [153]. Copyright 2023, nature.

A remarkable development in bioengineering, cell chips provide platforms for the high-throughput, controlled cultivation and study of living cells [153]. Usually, these chips are made up of tiny chambers or wells where cells can be seeded and grown in exact circumstances. The main use of cell chips is to simulate in vivo conditions in order to investigate how cells react to different stimuli, such as chemicals, medications, and environmental elements [154]. Cell chips are used by researchers to learn more about the behavior of cells, signaling pathways, and the effects of outside factors on cellular processes. These chips play a key role in drug discovery by enabling the screening of putative therapeutic compounds, evaluation of drug toxicity, and investigation of the effectiveness of prospective medications on cell types. Moreover, cell chips support stem cell production and manipulation, which advances studies in regenerative medicine. Because this technique makes it possible to systematically explore cellular responses in a controlled microenvironment, it holds potential for improving our understanding of cell biology, disease processes, and personalized therapy [155]. The Figure 6 depicts the microfluidic chip.

Revolutionary solutions in remote patient monitoring have been made possible by engineering advancements in the field of biomedical sensors and monitoring. The creation of all-encompassing systems that are not limited by geography has been made possible by the integration of various sensors, such as blood pressure, temperature, and ECG monitors. By providing real-time insights into critical health metrics, these built systems enable healthcare providers to monitor patients remotely. These remote patient monitoring devices improve patient care, support early intervention, and prompt healthcare decisions, and can be used for everything from managing chronic diseases to post-surgery or post-hospitalization monitoring. The field of patient monitoring has changed as a result of the collaboration between engineering and healthcare, bringing in a new era in which patients can receive continuous, individualized treatment regardless of where they are [157].

## 4. Drug delivery systems

The term ‘drug delivery systems’ refers to devices that transport medications into or through tissue. These technologies include the delivery mechanism, like an injectable vaccine or a tablet that you take. Medicine delivery systems can also refer to how pharmaceuticals are ‘packaged,’ such as in a micelle or nanoparticle, which keeps the medicine from breaking down and enables it to enter the body where it is needed [158]. Drug delivery has come a long way in the last few decades, and more advancements are expected in the years to come. Biomedical engineers have made significant contributions to our knowledge of the physiological obstacles to effective drug administration as well as to the creation of several novel drug delivery techniques that are now being used in clinical settings [159]. Nevertheless, despite all this advancement, many medical therapies still have unacceptably high adverse effects. Drug interactions with healthy organs or tissues might result in side effects, which can make it more difficult to treat conditions including cancer, neurological disorders, and infectious infections. Sustained progress in this area will aid in both facilitating medication distribution that is specifically targeted and reducing the negative effects of the medications. Drug distribution offers a way to enhance patient adherence, boost effectiveness, and lessen ocular pharmaceutical side effects [160].

In the past, a variety of illnesses have been treated by drug delivery systems (DDSs). To treat illnesses, all medications rely on pharmacologic active metabolites, or pharmaceuticals [161]. Certain medications are intended to be inactive precursors that the body must change into active forms. The administration method affects their efficacy. In traditional drug delivery systems (also known as CDDSs), medications were often administered orally, nasally, inhaled, sublingually, or via injection. Conventionally administered medications caused injury to unaffected areas, were excreted early, had a slower rate of absorption, and required longer to cure the illness [162]. Numerous obstacles, including their enzymatic breakdown or pH imbalance, numerous mucosal barriers, off-target effects, and their rapid release that increased toxicity in blood, made them less effective. The development of the controlled-release drug delivery system was motivated by all these factors. Drug effectiveness is improved by this DDS evolution in several ways. Recently, DDSs have been designed to regulate the release of drugs. These altered DDSs released drugs into the sick areas under control using a variety of cutting-edge techniques. These tactics included hydrogel, matrix, osmotic pump, degradable substance, erodible material, and reservoir [163]. They all offered a means of delivering the medications to the targeted areas, such as organs, tissues, or cells. These methods frequently have medications accessible for a wide range of illnesses. These tactics didn’t work because of their poor impact on treating diseases, lesser distribution, less solubility, increased drug aggregation, and less target selection. Furthermore, the most costly, complex, and time-consuming procedure is medication development [164].

The more sophisticated varieties of NPs are called nanocarriers, and they include dendrimers, liposomes, peptide-based nanoparticles, carbon nanotubes, quantum dots, polymer-based nanoparticles, inorganic vectors, lipid-based nanoparticles, hybrid NPs, and metal nanoparticles [165]. These days, the field of using nanoparticles for tissue micro-engineering, drug delivery, microfluidics, biosensors, and microarrays for the targeted treatment of diseases is expanding. Although less effective, nanoparticles can treat cancer by eliminating only the malignant cells. The physicochemical characteristics of nanocarriers are what make them unique; they enhance the solubility, clearance, degradation, targeting, theranostics, and combination therapy of the medications [166]. Numerous diverse types and configurations of protein-based podiums, including multiple protein coops, nanoparticles, hydrogels, films, microspheres, tiny rods, and minipellets, are organized to transport medication. Drug transportation is a characteristic shared by all proteins, including ferritin-protein coop, the small heat shock protein (sHsp) cage, viral capsids generated from plants, albumin, soy and whey protein, collagen, and proteins embedded in gelatin [167].

### 4.1. Designing and development of drug delivery system

#### 4.1.1. Integrating computational modeling

Engineering simulation tools have become indispensable in DDS design and optimization. Computational models can predict drug release profiles, simulate transport mechanisms, and assess the impact of various factors on drug delivery, guiding the development of more effective DDS [168]. A new tool for overcoming the limitations and issues with conventional drug research and discovery techniques is computational simulation. An almost realistic representation that can hasten the process of finding and developing new drugs is a computational simulation. Throughout practically every phase of the drug development process, computational model tools have been widely utilized. Target identification and characterization, which entails figuring out a prospective therapeutic target’s function and relevance in the disease, is the first step in the early stages of drug discovery [169]. The molecular mechanism of the therapeutic target is characterized when it has been identified. Among the computational techniques used to narrow down a large number of potential targets to a select few known active targets are pharmacophore mapping and inverse docking. A good target should meet clinical and commercial objectives while also being safe and effective.

#### 4.1.2. Optimizing drug formulations

Engineering principles have guided the formulation of drugs to improve their stability, solubility, and permeability. This has led to the development of novel formulations, such as liposomes, nanoparticles, and prodrugs, that enhance drug delivery and efficacy [170]. In order to improve the effectiveness and safety of therapeutic agents, optimizing drug formulations is a crucial aspect of pharmaceutical development. The chemical characteristics of the active pharmaceutical substances, their bioavailability, and the intended pharmacokinetic profiles are just a few of the many variables that must be carefully taken into account during this complex procedure. The art and science of medication formulation involves scientists and researchers working to create the best possible solubility, stability, and controlled release [171]. The objective is to create formulations that maximize therapeutic effectiveness while reducing the possibility of side effects, ensuring precise medication delivery to the intended target areas within the body. Novel delivery systems and nanotechnology, among other advancements in pharmaceutical sciences, are contributing factors to the continuous search for better drug formulations. Pharmaceutical scientists work to overcome obstacles related to drug solubility, bioavailability, and patient adherence by optimizing drug formulations, thereby opening the door for more efficient and patient-centered therapeutic interventions [172].

#### 4.1.3. Designing delivery devices

Engineers have devised ingenious devices for administering drugs, such as implantable pumps, microneedles, and transdermal patches. These devices provide precise control over drug delivery, ensuring optimal therapeutic outcomes [173]. One of the most important aspects of developing a drug delivery system (DDS) is designing delivery devices (DDD), which is where medical innovation and engineering creativity meet. In order to ensure that pharmaceutical compounds are administered precisely and to achieve the overall goal of maximizing therapeutic outcomes while minimizing potential adverse effects, delivery devices must be designed with great care. Engineers have revolutionized DDS by creating innovative techniques and high-tech gadgets that surpass conventional medication delivery strategies [174]. These delivery systems cover a wide range of advances, such as wearable technologies, implanted devices, and controlled-release mechanisms, all of which are intended to improve treatment efficacy and patient compliance. Through the application of engineering principles, DDD not only makes controlled drug release possible, but also tackles issues like targeted delivery, biocompatibility, and patient convenience. A new age in drug delivery is being ushered in by the convergence of engineering and medical sciences in the design of delivery systems. These developments have the potential to completely transform modern medicine and greatly enhance patient care [175].

The illness process involves a molecular target, and in order for a therapeutic intervention to be effective, the target must be modulated [176]. To optimize and validate the identified targets, researchers employed a variety of advanced computational techniques, including sequence-based approaches, pharmacophore modeling, de novo design, virtual library design, quantitative structure – activity relationship, and molecular docking. Recently, computational modeling has been increasingly important in the design and development of nano formulations, leaving its mark on subsequent stages of drug discovery and development, including preclinical trials [177]. The integration of biomedical sciences and engineering has resulted in revolutionary developments in DDS. Pharmaceutical distribution can be made more precise, effective, and targeted with the use of engineered solutions.

The engineering-driven developments in DDS that are showcased in Table 4 highlight the variety of uses and advantages that result from the application of engineering concepts in the healthcare field. Every entry describes a particular breakthrough, both the technical basis and the resulting enhancements in medication administration. The table gives a summary of how various innovations, such as controlled-release technologies and targeted nanocarriers, are strategically used to improve treatment outcomes, reduce side effects, and completely change the field of pharmaceutical interventions.

**Table 4.** Fda-approved drug products of injection delivery [287].

S.NO	Drug Name	Approval Date	Active Ingredients	Composition/Type	Company	Indication
1.	Doxil	1995	Doxorubicin hydrochloride	HSPC, cholesterol and PEG	Janssen	Ovarian Cancer; Sarcoma; Myeloma
2.	Ambisome	1997	Amphotericin B	HSPC,DSPG, cholesterol, and amphotericin B	Astellas	Fungal infection
3.	Depocyt	1999	CytarabineCytarabine	Cholesterol, Triolein, DOPC and DPPG	Pacira	Lymphomatous
4.	Exparel	2011	Bupivacaine	DOPC and DOPE	Pacira	Local anesthetic
5	Marqibo kit	2012	Vincristine Sulfate	Cholesterol and eggs sphingomyelin	Talon	Acute lymphoblastic leukemia
6	Onivyde	2015	Irinotecan hydrochlorine	DSPC, MPEG-2000-DSPE	Ipsen	Adenocarcinoma of the pancreas
7	Generic drugs					
8	Doxorubicin hydrochloride	2013	Doxorubicin hydrochloride	DSPC and cholesterol	Sun pharma	Ovarian cancer; sarcoma
9	Doxorubicin hydrochloride	2017	Doxorubicin hydrochloride	DSPC and cholesterol	Dr Reddys	Ovarian cancer; sarcoma
10	Microsphere					
11	Lupron Depot	1989	Leuprolide Acetate	PLGA	Abbvie	Advanced prostatic cancer
12	Sandostatin Lar	1998	Octreotide acetate	PLGA	Novartis	Acromegaly
13	Trelstar	2000	Triptorelin pamoate	PLGA	Allergen	Advanced prostate cancer
14	Definity	2001	Perflutren	DPPA, DPPC and MPEG-5000-DPPE	Lantheus	Ultrasound contrast agent
15	Risperdal Consta	2003	Risperidone	PLG	Janssen	Schizophrenia; Bipolar I Disorder
16	Vivitrol	2006	Naltrexone	PLG	Alkermes	Alcohol dependence
17	Bydureon	2012	Exenatide synthetic	PLGA	AstraZeneca AB	Type 2 diabetes
18	Signifor Lar	2014	Pasireotide pamoate	PLGA	Novartis	Acromegaly
19	Lumason	2014	Sulfur hexafluoride lipid-type microspheres	DSPC and DPPG-Na	Bracco	Ultrasound contrast agent
20	Bydureon Bcise	2017	Exenatide	PLGA	AstraZeneca AB	Type 2 diabetes
21	Triptodur Kit	2017	Triptorelin pamoate	PLGA	Arbor	Central precocious puberty
22	Atridox	1998	Doxycycline hyclate	PLA	Tolmar	Chronic adult periodontitis
23	Eligard	2002	Leuprolide acetate	PLGA(Atrigel)	Tolmar	Advanced prostate cancer
24	Abraxane	2005	Paclitaxel	Protein nanoparticle	Protein nanoparticle	Metastatic Breast Cancer; Non-Small Cell Lung Cancer
25	Somatuline Depot	2007	Lanreotide acetate	Nanotube	Ipsen	Acromegaly
26	Zyprexa Relprevv	2009	Olanzapine pamoate	Microcrystal	Eli lilly	Schizophrenia
27	Invega Sustenna	2009	Paliperidone palmitate	Nanocrystal	Janssen	Schizophrenia
28	Feraheme	2009	Ferumoxytol	carbohydrate-coated iron-oxide nanoparticle	Amag	Iron deficiency anemia
29	Sustol	2012	Granisetron	Ortho ester (Biochronomer™)	Heron	Nausea and vomiting
30	Abilify Maintena	2013	Aripiprazole	Nanocrystal	Otsuka	Schizophrenia
31	Ryanodex	2014	Dantrolene sodium	Nanocrystal	Eagle	Malignant hyperthermia
32	Invega Trinza	2015	Paliperidone palmitate	Nanocrystal	Janssen	Schizophrenia
33	Aristada	2015	Aripiprazole Lauroxil	Nanocrystal	Alkermes	Schizophrenia
34	Sublocade	2017	Buprenorphine	PLGA	Indivior	Moderate to severe opioid use disorder
35	Intralipid	1975	Soybean Oil	Fat Emulsion	Fresenius	Parenteral nutrition
36	Cleviprex	2008	Clevidipine	Lipid emulsion	Chiesi	Reduction of blood pressure
37	Perikabiven	2014	Amino acids	Lipid emulsion	Fresenius	Parenteral nutrition
38	Smoflipid	2016	Fish oil	Lipid emulsion	Fresenius	Parenteral nutrition
39	Cinvanti	2017	Aprepitant	Lipid emulsion	Heron	Acute and delayed nausea and vomiting
40	Onpattro	2018	Patisiran	Lipid nanoparticle	Alnylam Pharmaceuticals	Polyneuropathy caused by hereditary transthyretin-mediated amyloidosis
41	Vyondys 53	2019	Golodirsen	Phosphorodiamidate morpholino oligomer	Sarepta Therapeutics	Duchenne muscular dystrophy
42	Givlaari	2019	Givosiran	Lipid nanoparticle	Alnylam Pharmaceuticals	Acute hepatic porphyria
43	Doxil	2019	Doxorubicin hydrochloride	Liposome	Teva Pharmaceuticals	Ovarian cancer, AIDS-related Kaposi sarcoma, multiple myeloma
44	Myocet	2019	Doxorubicin hydrochloride	Liposome	Teva Pharmaceuticals	Breast cancer
45	DepoDur	2020	Morphine sulfate	Liposome	Pacira BioSciences	Pain management
46	Abraxane	2020	Paclitaxel	Albumin-bound nanoparticle	Bristol-Myers Squibb	Breast cancer, non-small cell lung cancer, pancreatic cancer
47	Onivyde	2020	Irinotecan hydrochloride	Liposome	Ipsen Biopharmaceuticals	Pancreatic cancer
48	Marqibo	2020	Vincristine sulfate	Liposome	Spectrum Pharmaceuticals	Philadelphia chromosome-negative acute lymphoblastic leukemia
49	DaunoXome	2020	Daunorubicin hydrochloride	Liposome	Galen US	Kaposi sarcoma
50	Visudyne	2021	Verteporfin	Liposome	Bausch Health Companies	Age-related macular degeneration
51	Lipo-Dox	2021	Doxorubicin hydrochloride	Liposome	Sun Pharma Global	Ovarian cancer, AIDS-related Kaposi sarcoma, multiple myeloma
52	Lipodox 50	2021	Doxorubicin hydrochloride	Liposome	Sun Pharma Global	Breast cancer
53	DepoCyt	2022	Cytarabine	Liposome	Pacira BioSciences	Lymphomatous meningitis
54	Vyxeos	2023	Daunorubicin and cytarabine	Liposome	Jazz Pharmaceuticals	Acute myeloid leukemia

### 4.2. Smart drug delivery systems (SDDS)

Smart drug delivery systems are state-of-the-art technologies that are intended to precisely, efficiently, and frequently with controlled release kinetics deliver drugs or therapeutic substances to specified target areas within the body. These systems are referred to as ‘smart’ because they have the ability to release the drug payload in response to both internal and external stimuli, such as variations in pH, temperature, enzyme activity, or light. The state-of-the-art in drug delivery is represented by SDDS, which are made to react intelligently to certain stimuli and maximize therapeutic effects. Different categories of stimuli-responsive SDDS have been examined in the discussion that follows [178]. Stimuli-responsive systems include Temperature-Responsive SDDS, where drug release is modulated by changes in temperature; Redox-Responsive SDDS, which respond to variations in the cellular redox environment; PH-Responsive Systems, capable of altering drug release based on pH levels in different body compartments; Light Responsive SDDS, which can release medication in response to exposure to light; Enzyme-Responsive Drug Delivery Systems, which exploit enzymatic activity for triggered drug release; and Magnetic-Responsive Drug Delivery Systems, utilizing magnetic fields to control drug release at targeted sites [179]. With controlled and triggered release mechanisms, this wide range of stimuli-responsive methods promises greater therapeutic efficacy while minimizing side effects, demonstrating the diversity and accuracy that may be accomplished in drug delivery [180].

#### 4.2.1. Stimuli responsive SDDS

To improve therapeutic efficacy and lessen side effects, SDDS were created with the payloads being released to the target region in a more regulated manner. Systems that are stimulus-responsive react to both internal and exterior inputs [181]. The magnetic, electric, thermal, and acoustic forms of energy are examples of external stimuli. The redox status, pH, and temperature of the system, as well as biochemical elements like certain enzymes, urea, glucose, and the morphine responsive system, as well as particular medical problems like cancer and inflammatory response system, are examples of internal stimuli [182].

##### 4.2.1.1. Temperature responsive SDDS

The medications will be released quickly as the temperature climbs over the physiological temperature since thermo sensitive polymers are made to hold their payloads around that temperature. These systems are appropriate for the regulated administration of genes and medications. Temperature-sensitive polymers go through sol-gel transitions, and even slight variations in body temperature can be seen in the solubility changes [183]. Pathologic tissues are hotter than normal tissues when it comes to clinical diseases like inflammation and cancer [184]. When a temperature shift occurs, the temperature responsive SDDS are triggered since they can detect it. Using temperature responsive SDDS has the benefit of allowing for the application of external or internal stimuli. The medication is also released into the tumor microenvironment with the aid of temperature generation induced at the malignant location. For instance, the thermosensitive polymer poly (N-isopropyl acrylamide) (PNIPAAm) has a lower critical solution temperature of 32°C and is brought to physiological temperature by surfactants or additives [185]. The solution gets hazy and gels when the temperature goes above 27°C. Utilizing thermosensitive polymers, a variety of thermo-responsive nanocarrier forms, including hydrogels, microbeads, micelles, polymeric nanotubes, core-shell thermo-responsive nanoparticles, and layer-by-layer constructed nanocapsules, have been created and are being employed as SDDS [186].

##### 4.2.1.2. Redox responsive SDDS

**Figure 7.**
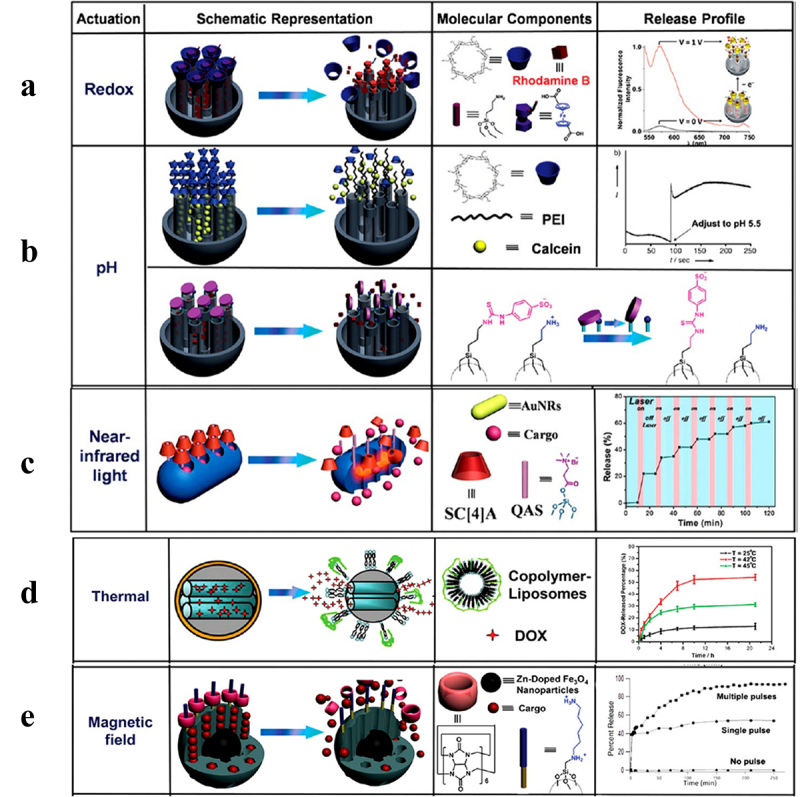
Summary of operation of stimuli-responsive polymers in redox, pH, UV light and near-ir light and magnetic field with permission, this image has been reproduced [186]. Copyright, 2019 ACS.

Due to its close relationship to numerous diseases, redox-sensitive drug delivery systems have drawn a lot of attention and have been thoroughly explored (Figure 7a). The intracellular drug release feature of the redox-sensitive delivery method is an additional benefit. This approach is designed to take advantage of the variations in the redox state of tissues [187]. For the most part, redox-responsive SDDS are intracellular glutathione-dependent systems. Another important class of drug delivery methods in redox-sensitive systems are oxidation-responsive drug delivery systems, which primarily rely on ROS, primarily H2O2 and -OH radicals. ROS are ubiquitous in tissues and are linked to a number of clinical diseases, including inflammation, heart and nerve damage, and arteriosclerosis. The sulfide-containing drug delivery systems, such as poly (propylene sulfide), selenium-containing, ferrocene-containing, boronic ester groups, etc., are among these classes [188].

##### 4.2.1.3. PH responsive systems

In most disease-related applications, medication delivery to specific areas is necessary to achieve maximal efficacy while minimizing harm. The pH of organs, organelles, and tissues varies, making pH-responsive systems a more suitable option and a widely used SDDS (Figure 7b) [189]. Predefined release rates and exact delivery options are also provided by this system. Every pH-responsive polymer that is utilized has a pendant group acting as a donor or acceptor of protons, such as sulfonic and carbonic acids, or basic, such as ammonium salts. These carriers are able to track minute variations in the pH of malignant or inflammatory areas. In general, the pH of the tumor microenvironment is lower (7.0) than that of the normal microenvironment (pH 7.4). The ionization of pendant acidic or basic groups brought on by variations in the ambient pH results in cross-linking and modifications to the swelling characteristics of the polymer. Combining pH-responsive systems with additional stimuli, like temperature or redox stimuli, increases their precision and accuracy. This is known as a multifactor-responsive drug-release system [190]. The pH of the solution the polymer is exposed to determines the kind of drug release that occurs. The polymer shrinks or swells in response to the pH change. For instance, the protonation of acidic groups causes polyacidic polymers to grow at high pH and shrink at low pH. Ionization of basic groups rises with increasing pH in the case of polybasic polymers [191].

##### 4.2.1.4. Light responsive drug delivery systems (LRDDS)

Light is regarded as one of the most interesting external stimuli for controlled drug release because it has an on/off switching pulsatile behavior that allows for remote drug release with extreme temporal and spatial precision(Figure 7c) [192]. LRDDS are mostly used in photodynamic therapy (PDT), a combination therapy that uses light and photoactivatable photosensitizer in the presence of tissue oxygen. Through an intravenous injection of the photosensitizer, PDT produces radical oxygen species (ROS) from the tissue oxygen through light-mediated activation. Because of the cellular necrosis that these ROS produce, PDT is a prime choice for cancer therapy [193]. Photoreactions in PDT are triggered by UV or visible light. Only topical treatments applied to the skin or mucosa contain UV or visible light. The majority of applications in biomedicine use near-infrared (NIR) light, which falls between 650 and 900 nm in wavelength, for deeper light penetration, that is, more than a few millimeters [194]. Recently, NIR-based photosensitizers for photodynamic therapy were described by Xu et al. [195] in 2019.

##### 4.2.1.5. Enzyme responsive drug delivery systems

A novel kind of drug delivery system called an enzyme activated drug delivery system uses enzyme reactions to regulate the release of the drug. Enzyme-responsive drug delivery systems are being developed using a variety of polymers and nanomaterials [196]. One benefit of this approach is its high level of specificity. The majority of physiological and metabolic processes involve the involvement of enzymes including protease, phospholipase, lipase, and glycosidase. These enzymes are used in biocatalytic action in inflammatory or malignant locations to achieve enzyme-mediated drug cargo release. The medications will be released at the target sites by site-specific enzymatic cleavage of the nanocarriers bearing payload coupled with them through encapsulation or covalent bonding [178]. The drug-release system is triggered by several enzymes. Proteases belong to several groups and are engaged in different physiological and pathological circumstances such as invasion of tumors, remodeling of tissues, and wound healing. The most often utilized protease enzymes in controlled medication release are matrix metalloproteinases (MMPs), protein kinase C-alpha, and uPA (urokinase type plasminogen activator) [197]. Figure 7 depicts the summary of operation of stimuli-responsive polymers in redox, pH, UV light and near-IR light and magnetic field.

##### 4.2.1.6. Magnetic responsive drug delivery systems

Systems for delivering drugs that respond to magnetic fields offer a noninvasive method of controlling the carriers’ movement in relation to their objectives (Figure 7e). This facilitates the payloads’ release from the system when it is programmed to be exposed to external magnetic fields. Numerous distinct magnetic characteristics are displayed by the most widely used core/shell magnetic nanoparticle [198]. The medication plus a ferromagnetic carrier that is pharmaceutically stable make up the complex. Magnetic nanoparticles (MNPs) can have a variable surface-to-volume ratio depending on how they are made. A higher surface-to-volume ratio offers a large number of active sites for biomolecule conjugation. By providing a localized external magnetic field, this enables precision in design and engineering to achieve their smart features, such as improved delivery, specificity in the release site, and elevated blood half-life [199].

Moreover, these nano scaled MNPs become more susceptible to an external magnetic field when they are enclosed in colloidal carriers such solid nanoparticles, liposomes (Magneto liposomes), and micelles. The building of multi-stimuli-responsive drug delivery systems that can influence pH, redox, and enzymes is also made possible by these magnetic nanoparticles. For instance, doxorubicin-encapsulated gelatin treated with cystamine demonstrated improved drug target selectivity, excellent biocompatibility, and enhanced electrophoretic potential [200]. Drug delivery systems (DDS) are essential to contemporary medicine because they allow medicinal substances to be released under precise control, improving treatment outcomes while reducing side effects. Engineering concepts have transformed DDS development, resulting in creative methods that are changing the medical field.

### 4.3. Brain drug delivery system using nanoparticles

The blood – brain barrier (BBB) is compromised in the most pathological conditions of disorders such strokes, seizures, multiple sclerosis, AIDS, diabetes, gliomas, Alzheimer’s disease, and Parkinson’s disease. The pathological remodeling of the protein complex in intra-endothelial junctions is a major factor contributing to the collapse of the blood – brain barrier [201]. Normally, the blood-brain barrier blocks the passage of blood macromolecules and micromolecules to preserve blood-brain equilibrium. Drugs that pass through the blood-brain barrier accumulate less in the intracerebral area of the brain and have lower bioavailability, which makes treating brain illnesses impossible. Consequently, the best drug delivery system (DDS) is one that is based on cell membranes, viruses, or exosomes that is made to be lesion-targeting, BBB penetrable, and generally safe [202]. The intranasal medication carriage system supported by nanocarriers is a commonly employed method for treating brain illnesses. Currently, at an advanced stage, medications that are not well distributed to the brain can be put into a system based on nanocarriers. This system would work in concert with the BBB’s endothelial micro vessel cells and the nasal mucosa to extend the time that drugs take to absorb into the body, as well as the olfactory nerve fibers to encourage straight nose-to-brain delivery, which will increase drug absorption in the brain parenchyma through the secondary nose to blood to brain pathway.

Viral vectors, exosomes, brain permeability enhancers, delivery via active transporters in the blood-brain barrier, changing the route of administration, brain-specific nanoparticles, and imaging/diagnostics in pathological states are the current tactics being used [203]. Among the neurodegenerative disorders that affect the older population most quickly on the rise is Alzheimer’s disease. Nanotechnology-based techniques are used in the treatment of several diseases by drug delivery based on nanotechnology. Liquid crystals, polymeric nanoparticles, liposomes, solid lipid nanoparticles, nano-emulsions, and micro-emulsions are all employed in the treatment of Alzheimer’s disease [167]. As the second most prevalent neurological condition, Parkinson’s disease has challenges with accurate medication distribution for both diagnosis and therapy. The most interesting issue is that levodopa, the traditional anti-Parkinson’s medication, has poor brain transfer and low absorption [204]. Nanotechnology steps up to the plate with clever ideas to overcome this problem. Treatments for Parkinson’s disease involve the use of a variety of nanoparticles, including liposomes, metal nanoparticles, quantum dots, organic nanoparticles, cerium oxide nanoparticles, and gene therapy [205].

### 4.4. Role of nanocarriers for cancer therapy

Surgery, chemotherapy, radiation therapy, hormonal therapy, and targeted therapy are all used to treat breast cancer effectively. On the other hand, interest in using nanotechnology to treat breast cancer has grown recently. Drugs are delivered to the precise target spot using a variety of organic and inorganic nanocarriers. Nanocarriers facilitate targeted drug distribution and increase the hydrophobicity of anticancer medications [206]. Polymeric, liposome, and solid lipid nanocarriers are examples of organic nanocarriers; magnetic, quantum dots, and carbon nanotubes (CNTs) are examples of inorganic nanocarriers; both types of nanocarriers exhibit excellent outcomes in the treatment of cardiac disorders. Numerous lung conditions, including cancer, emphysema, TB, cystic fibrosis, and asthma, can be cured with the use of nanoparticles [207].

**Figure 8.**
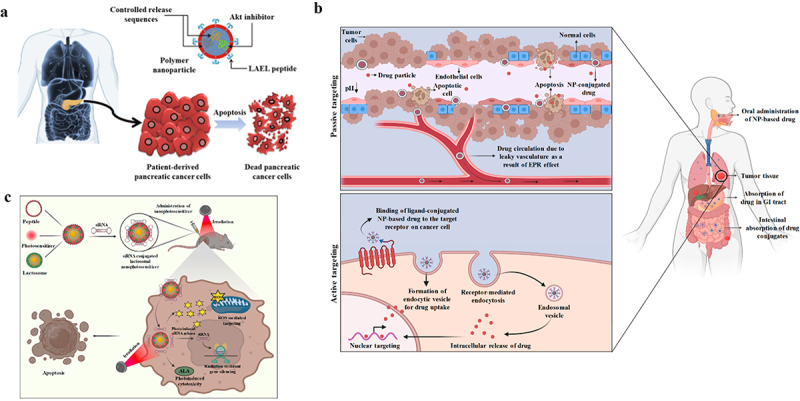
(a) Schematic diagram of self-assembling genetically engineered polymeric nanoparticles formed by elastin-like recombinamers (ELRs), carrying a small peptide inhibitor of the protein kinase Akt, in both PANC-1 and patient-derived pancreatic cancer cells (PDX models)(adapted with permission from ref [208], copyright, 2021 ACS). (b) Mechanism of passive and active targeting of np-drug conjugates. (c) Lactosomal nanophotosensitizer mediated photodynamic treatment (adapted with permission from ref [289], copyright, 2023 MDPI).

Juan et al. has tested self-assembling genetically engineered polymeric nanoparticles formed by elastin-like recombinamers (ELRs), carrying a small peptide inhibitor of the protein kinase Akt, in both PANC-1 and patient-derived pancreatic cancer cells (Figure 8a) [208]. Multiple nanowires found in sensor test chips have the capacity to alter individual cells and identify oncogenic cell biomarkers, which aid in the early detection of cancer even with a small blood sample. In order to monitor and control cancer cells as well as pinpoint the genetic and biomolecular targets for upcoming treatments, nanoscale probes are utilized to watch intricate molecular and cellular activities in real time. Furthermore, it is anticipated that the application of nanolabels will provide improvements in high-throughput screening and gene expression research. Because of their ability to change into a broad variety of forms and sizes during the production process, nanoparticles are useful for usage as drug carriers in products like immunoconjugates, polymer drug conjugates, and liposomes. Effective targeting of cancer cells with drug delivery via nanoparticles is crucial for improved therapeutic efficacy, decreased dose-limited toxicity, and safeguarding healthy cells from systemic toxicity. Anti-cancer drugs should be delivered to the tumor location using nanoparticles in a method that allows them to bypass all phagocytic barriers and enter the tumor tissues with the least amount of blood volume or activity loss [209].

Tissue morphology at the microscopic level from a biopsy or surgically removed tumor is a major factor in the diagnosis of cancer. Hematoxylin is used for histopathology examination, while electron microscopy is used to assess the histomorphology of the tissues stained with eosin [210]. Furthermore, bioconjugated particles are being created for the earlier detection of cancer in bodily fluids, such as blood and serum [211]. As they are frequently present in extremely low concentrations, the application of nanoparticles for the detection and evaluation of circulating cancer cells or their biomarkers present in blood and serum samples is another promising area of research [212]. However, combinatorial employment of semiconductor quantum dots and magnetic nanoparticles may be able to enhance the capacity to capture and analyze these rare circulating cancer cells. Nanotechnology may therefore profit from the unique architecture, vascularity, antigenicity, biomarkers, and microenvironment of tumors for the therapy and surveillance of cancer [213]. Figure 8 illustrates cutting-edge advancements in nanoparticle-based drug delivery and targeted therapy for pancreatic cancer.

### 4.5. Microneedle patch for painless vaccinations

**Figure 9.**
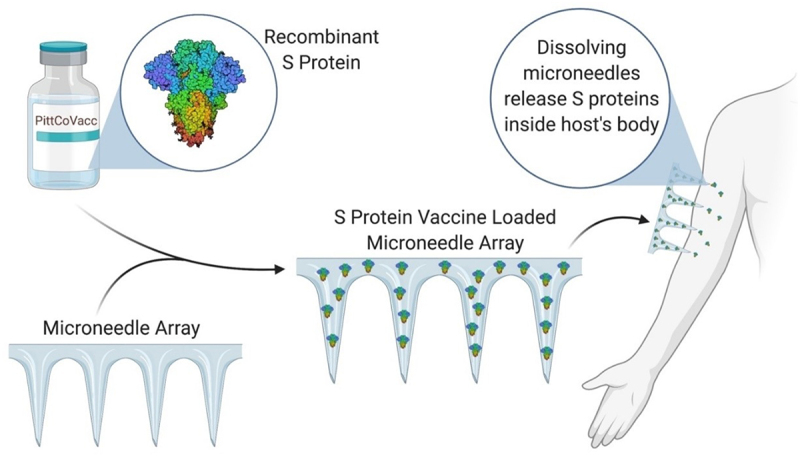
Schematic representation of microneedle assisted COVID-19 vaccine delivery platform. Adapted with permission from ref [215], Copyright, 2022 ACES).

One novel way to administer drugs through the skin is using micro needle arrays. Numerous tiny needles, each much thinner than a human hair strand, can be made to hold a medication in these arrays. Because the needles are so tiny, they can deliver drugs painlessly even though they pierce the skin without reaching the nerves. To deliver vaccines, scientists are creating a patch with a variety of dissolvent micro needles. Patients could use these patches at home because they are simple to use, don’t need to be refrigerated, and don’t require any particular disposal techniques. Low-resource regions might not have many healthcare professionals or suitable storage facilities for conventional, refrigerated medications, thus this technique could be very helpful in those areas [214]. The creation of advanced delivery systems, like MN, can lower the overall dosage of medication while simultaneously increasing delivery efficiency. The rate at which needles dissolve is one factor that can alter the bioavailability rate. Similarly, altering the size and shape of the needle can aid in boosting the drug’s deeper transdermal release. Additionally, using water-soluble formulations or placing biopharmaceutical agents far from the basement in close proximity to needle tips may increase the bioavailability of drugs. In addition, distinct geometrical parameters that are used in the creation and transportation of different compounds into the deeper layer of cutaneous tissue, such as needle height, needle tip radii, and needle side wall thickness, are all the same. Recently, a range of needles smaller than 500 µm has been created for MN devices, allowing for effective administration across the epidermal barrier. The ability to load both large- and small-sized molecules into MN devices, including proteins, nucleic acids, vaccines, hormones, nanoparticles, and virus-like particles, is one of the system’s most notable benefits. Because MN devices provide painless injections, they improve patient compliance while reducing the risk of in situ infections from conventional needle use. Although the MN system might aggravate skin irritation or trigger allergic reactions, it has great potential as a substitute for conventional approaches. Advances in MN device development have raised aspirations for employing fewer skilled workers globally by opening options for self-health administration. Vaccines were long thought to be the only effective treatment, but the return of modified SARS-CoV-2 strains (such variant B.1.617) and their capacity to re-infect survivors raise the possibility that the virus will eventually become endemic and experience sporadic flare-ups similar to the seasonal flu. Nonetheless, vaccination remains a potentially useful tool for managing certain diseases, such as the flu. Newer delivery methods, like PittCoVacc, a recombinant protein subunit vaccine targeting SARS-CoV-2 that is delivered by a microneedle array (MNA) (Figure 9), have made it possible to administer vaccines more frequently and conveniently [216].

### 4.6. Robotic pill for oral drug delivery of complex drugs

Certain disorders, like Crohn’s disease and diabetes, are managed using self-injections. Because the medications used to treat these illnesses are frequently complicated and readily metabolized, oral administration of the medication may be necessary. However, because of the frequency of injections and the risk of needle stick injuries, self-injection might provide challenges for patients. Scientists and engineers are working on a robotic pill that can hold complex liquid medications as an alternative to self-injection. This pill travels to the stomach after being swallowed, where the medication is injected into the stomach tissue [217]. A drug delivery robot that can administer several biotherapeutics for various purposes is called a robotic pill (RP). The RP is an enteric-coated mechanical robotic auto-injector that is housed in a typical pharmaceutical (methylcellulose) capsule shell to protect it from dissolving and discharging in the stomach’s acidic environment. A hollow, dissolvable needle with a specific dosage of the sterile biotherapeutic (payload) is placed into a micro syringe that is connected to a self-inflating balloon that is folded. The enteric coating and capsule shell dissolve in the small intestine due to a pH shift, exposing the RP to intestinal fluid. This sets off a chemical reaction that causes the balloon to rapidly inflate. This allows the dissolvable needle containing the medication payload to be injected into the intestinal wall while the micro syringe is positioned perpendicular to the small intestine’s long axis. The injection into the intestinal wall should not cause any discomfort because the intestine is insensitive to strong nociceptive stimuli. In earlier studies, we demonstrated that the RP can consistently provide bio therapeutics with great bioavailability in models of pigs and dogs.

## 5. Tissue engineering and regenerative medicine

Tissue engineering, rooted in biomaterials development, is the process of creating functional tissues using scaffolds, cells, and physiologically active chemicals. It operates within the broader field of regenerative medicine, which includes the study of self-healing mechanisms employed by the body to repair damaged tissues and organs, sometimes with the aid of external biological material. The terms ‘regenerative medicine’ and ‘tissue engineering’ have become almost synonymous as the focus of the profession shifts toward treating complex and often chronic diseases [218]. Tissue engineering is grounded in three fundamental principles that enable engineers to devise novel approaches for tissue regeneration and repair. The first principle involves the intentional use of biocompatible scaffolds that promote cell attachment and proliferation, serving as frames to guide the creation of new tissue. The second principle centers on the deliberate inclusion of cells, such as stem cells or other specialized types, arranged within the scaffold to promote tissue integration, differentiation, and growth. The third principle utilizes signaling molecules to control cellular activity, influencing the growth and performance of the tissue being created. These principles form the foundation of tissue engineering, offering a comprehensive framework for successfully replicating or replacing damaged tissues [219].

**Figure 10.**
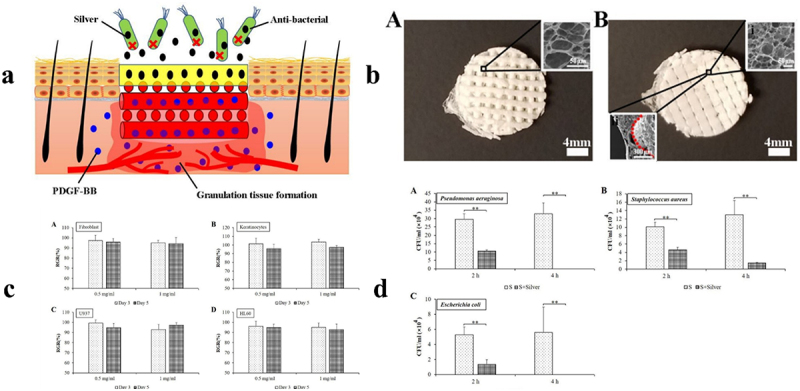
(a) Schematic diagram of bilayer 3D scaffold inspired by the structure of skin. (b) Physical characterization of 3D bilayer scaffold. (c) Biocompatibility test of silver-loaded 3D bilayer scaffold. (d) Silver-loaded 3D bilayer scaffold against Pseudomonas aeruginosa. (Adapted with permission from ref [290], copyright, 2019, Materials chemistry B).

**Figure 11.**
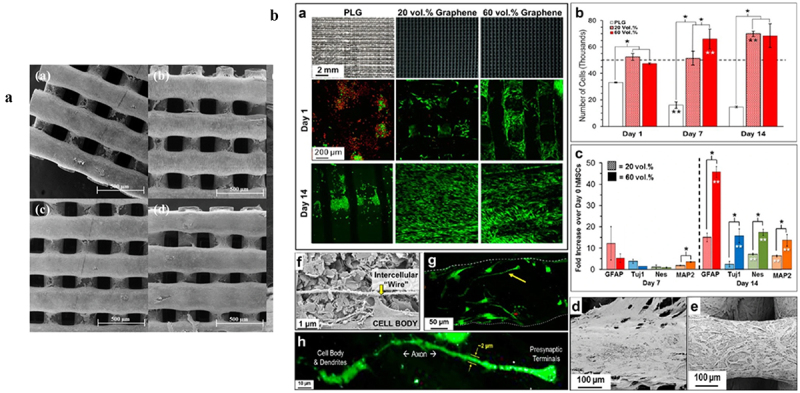
a. Schematic diagram of scaffold integrated graphene nanomaterials. b. (a) photographs (top row) and scanning laser confocal 3D-reconstruction projections of live stained (green) and dead stained (red) hMSCs on various scaffolds 1, 7, and 14 days after being seeded. (b) Number of hMSCs present on scaffolds. (c) Neurogenic relevant gene expression of cells on (d) 20 and 60 vol.% graphene 7 and 14 days after being seeded normalized to expression of day 0, unseeded hMscs (adapted with permission from ref [291], Copyright, 2015 ACS).

The field of tissue engineering and regenerative medicine (TERM) has seen significant progress in recent decades, driven by diverse research endeavors. These include biomaterial design, surface characterization and functionalization to enhance cell-material interactions and imaging. Various strategies have been proposed, such as directly implanting patient-isolated cells into defects, delivering bioactive molecules and growth factors targeting specific tissues, utilizing cell-free scaffolding biomaterials, and employing scaffolding structures with cells mimicking the extracellular matrix of natural tissues. The materials used in TERM methods encompass a wide range, from synthetic and natural polymers to inorganic biomaterials like metals, ceramics, and their hybrid combinations [220]. Polymers, whether natural or synthetic, offer diverse features such as biodegradation, mechanical qualities, high porosity, surface-to-volume ratio, and small pore size, making them suitable for engineering and regenerating various tissues [221]. Inorganic biomaterials, including metallic and ceramic materials, with distinct compositions and microstructures, have been proposed for mending or replacing impaired musculoskeletal components and addressing periodontal abnormalities [222]. Furthermore, organic-inorganic hybrid biomaterials, formed by blending organic and inorganic substances, result in multipurpose materials with customized mechanical, thermal, and structural stability characteristics. Achieving good phase compatibility, preserving the scaffolds’ porous structure, and maintaining their mechanical strength are crucial goals during the fabrication of composite scaffolds [223]. As shown in Figure 10 Silver nanoparticle had been integrated with 3D printed scaffold it found that silver nanoparticle as an antimicrobial agent along with ions release from silver also accelerated the tissue regenerations process. Similarly graphene nanosheets has been used in 3D printing for tissue regeneration in Figure 11.

Recent advances in tissue engineering and regenerative medicine have significantly impacted healthcare, introducing transformative technologies such as three-dimensional printing, which revolutionizes the production of intricate structures for customized implants and tissue scaffolds, ensuring precision during surgical procedures. Tissue bio fabrication, another groundbreaking development, holds the potential to design tissues for transplantation, expediting the quest for regenerative treatments for organ failure. Simultaneously, progress in biomaterials plays a crucial role in creating biocompatible structures vital for the success of synthetic tissues. Collectively, these advancements represent a major step forward in the pursuit of efficient tissue replacement and replication, showcasing the revolutionary possibilities that emerge when engineering meets medical necessity [224]. While tissue engineering and regenerative medicine have made remarkable progress, challenges persist. Successful tissue replication and replacement requires addressing complexities in the immune response and achieving robust vascularization. Innovative research at the intersection of engineering and healthcare, coupled with interdisciplinary collaboration, is essential to surmount these obstacles. Emerging trends in immunomodulation tactics and innovative methods for vascular network creation offer promising prospects for tissue engineering. Overcoming these challenges and leveraging new opportunities will be essential for maximizing the benefits of tissue engineering in clinical outcomes [225].

In the field of biomedical engineering, the development and evaluation of replacements in tissue engineering demands a deep understanding of the mechanical properties of tissues and biomaterials. Comprehensive assessment of tissue engineering advancements is facilitated by detailed measurements of mechanical properties, a crucial aspect in the rapidly evolving field of biomedical engineering [226]. For instance, in the realm of cartilage substitutes, contemporary technologies are integral. Novel protocols combined with state-of-the-art techniques, such as 3D bioprinting, enable the creation of complex cartilage structures resembling the original tissue. This not only showcases possibilities for customized solutions in patient-specific settings but also unveils the mechanical performance of alternatives. Biomechanical testing tools, incorporating innovations like wireless sensors and real-time data analytics, provide dynamic insights into the mechanical behavior of engineered tissues. Additionally, artificial intelligence algorithms in computer modeling facilitate the simulation of intricate mechanical interactions, aiding in the identification and prediction of mechanical properties under diverse conditions [227].

Therefore, Section 5 becomes a critical juncture in our exploration, where conventional wisdom converges with cutting-edge innovation. By employing appropriate procedures and a range of experimental and theoretical techniques, we enhance our understanding of mechanical characteristics, contributing to the ongoing narrative of revolutionary advancements in tissue engineering. The inclusion of these contemporary instances underscores the dynamic interplay between traditional approaches and new technologies, guiding us toward a future where precision and creativity converge in the pursuit of optimal mechanical performance in biological substitutes.

## 6. Biomedical robotics and automation

These days, robotic devices are used for a wide range of delicate surgical tasks, including minimally invasive and open procedures, replacing missing limbs, teaching individuals with learning disabilities, giving medication, teaching stroke patients, and performing neuro-rehabilitation therapy [228]. Medical robots used in various illnesses for diagnosis and intervention: It is crucial to stress that, to meet all the needs of a suitable technological health solution, specialized physicians, therapists, engineers, and scientists from many domains must work together flawlessly during the design of each bio mechatronics device. For instance, medical robots are used for intervention and diagnostics. In the context of healthcare, robotics is described as a system that can perform biomechatronic operations based on the analysis of signals from sensors to offer medical care, including the confirmation of medical diagnoses, the administration of therapies, the support of rehabilitation, the enrollment of patients in preventative programs, etc. The most popular specifications for medical robots include quality, safety, remote control, improved documentation access, and so on [229].

A revolutionary era in healthcare is being ushered in by biomedical robots and automation, which is redefining patient care and medical procedures. The incorporation of robotic technologies has led to previously unheard-of levels of accuracy and effectiveness in a range of medical procedures [230]. Traditional healthcare paradigms are being fundamentally altered by the effect of biomedical robots, from procedures that require precise accuracy to diagnostics that require quick and accurate assessments. Beyond procedural improvements, robotics is bringing about a paradigm shift in patient care through customized treatment plans, creative rehabilitation strategies, and assistive technology. This introduction lays the groundwork for a discussion of how these technological developments are redefining patient-centered care from the ground up, not only by changing processes [231].

Biomedical robotics is a field that is now revolutionizing healthcare through applications in surgery, diagnosis, and rehabilitation. In the field of minimally invasive operations, surgical robots have become crucial because they allow doctors to perform complex surgery with greater precision. One such example is the da Vinci Surgical System, which makes a variety of treatments easier, including heart interventions and prostate surgery [232]. Robots are essential to diagnostics since they help with imaging and sample collection. Autonomous robotic systems represent the integration of robotics and diagnostics in surgery, such as the iKnife for real-time tissue examination. Furthermore, gait training, physical treatment, and the restoration of motor function are all aided by rehabilitation robots. The EksoGT exoskeleton is one device that helps people with spinal cord injuries regain their mobility. These illustrations highlight the wide-ranging and significant uses of biomedical robotics, highlighting their critical role in transforming the face of contemporary healthcare [233].

Traditional methods of medical imaging and diagnostics have been completely transformed by the integration of robotics in the field of diagnostic automation. Robotic-assisted imaging instruments improve the precision and efficacy of diagnostic processes, leading to more accurate medical assessments. These cutting-edge tools, like automated imaging systems and platforms for robotic-assisted surgery, allow medical practitioners to perform complex diagnostic procedures with unmatched accuracy [228]. These tools help to lower the margin of error and speed up diagnostic procedures by utilizing automation. Notably, the combination of robotics and diagnostic technology improves the overall caliber of medical evaluations and can optimize processes, which will ultimately improve patient outcomes and make the healthcare delivery system more efficient. Medical robots have come a long way since the 1980s. Robotics has benefited medical professionals and patients more compared to other scientific fields, which was unthinkable decades ago. Examples include skilled treatment, effective surgery, and excellent patient care in a secure setting [234].

**Figure 12.**
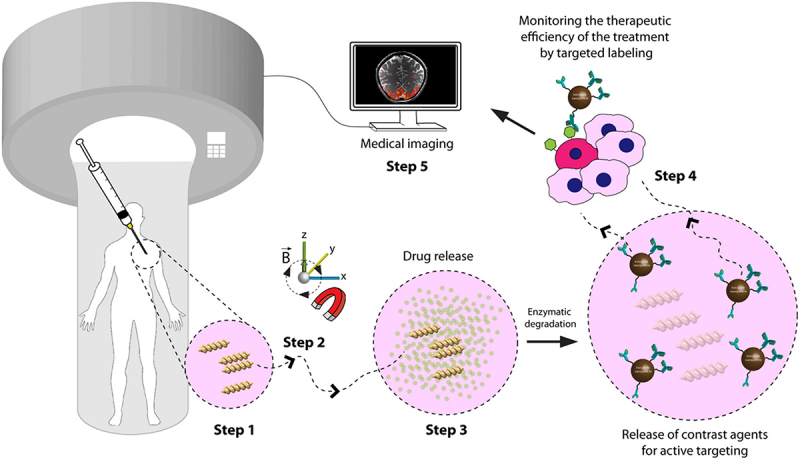
Envisioned theranostic application scenario of the 3D-printed, biodegradable microrobotic swimmers. Step 1: injection, step 2: active navigation and precise localization of the microswimmers at the site of medical intervention. Step 3: therapeutic intervention with controlled cargo release based on the pathological signal input at the tumor microenvironment. Step 4: complete biodegradation enables both the safe removal of the microswimmers and the release of diagnostic contrast agents. Step 5: antibody-modified magnetic contrast agents diffuse around to label the untreated tissue sites (adapted with permission from ref [238], copyright, 2019, ACS).

They support the delivery of more thoughtful and thorough treatment to patients, hastening the healing process. Augmented virtual telepresence, made possible by robotic technologies, enables users to visit remote areas and engage with local people, objects, and surroundings without physically visiting there. Similar to this, robot technologies have greatly advanced surgery; examples include the use of robots in neurosurgery and bone cutting [235]. Nevertheless, the main disadvantages of robots are their high cost and the need for surgeons to undergo extensive training tailored to certain tasks, which has prevented the technology from benefiting the world’s poor. Furthermore, one billion individuals, or 15% of the global population, live with a disability of some kind; the situation is particularly dire in poorer nations. Individuals with disabilities depend on their friends and family for assistance [236]. These people try to fill up the care gaps, but they also have jobs and other obligations. As a result, they are unable to offer services that cater to the requirements of people with disabilities. Conversely, the labor shortage has left healthcare staff overworked, with many more patients in need of care than there are personnel available. Furthermore, the problem has been made worse by COVID-19’s susceptibility to impact on them. As a result, robotics technology holds great promise for closing care gaps and assisting medical practitioners. Exoskeletons and other assistive robots are trained to regain grip, walking, and other body functions. Additionally, exoskeletons are utilized to help people who are chronically immobilized rehabilitate their upper and lower extremities [237]. A soft biodegradable microswimmer with both therapeutic and diagnostic release capabilities could be used for semiautonomous, minimally invasive microrobotic operations. In these operations, the therapeutic intervention’s effectiveness could be tracked by using magnetic contrast agents to locate the remaining target cells. The microswimmers could be implanted close to a tumor in a potential theranostic application (Figure 12). From there, the external magnetic fields might be used to remotely control them via microtracks, allowing for exact localization, steering, and navigation to the tumor with the least amount of harm to the delicate tissues nearby [238].

Biomedical robots and AI combine to create a game-changing combination that greatly improves these sophisticated systems’ ability to adapt and make decisions. AI algorithms enable biomedical robots to quickly digest large volumes of data and make well-informed judgments, enhancing their performance in a range of medical applications [239]. By combining these two technologies, robotic systems become more flexible and can react quickly to changing and complex situations, which increases their overall accuracy and efficiency. However, there are difficulties with integration. Implementation of biomedical robots is limited by issues like cost and accessibility. Reaching the full potential of these technologies requires overcoming these obstacles. Biomedical robotics appears to have a bright future ahead of it, with anticipated advances in autonomous systems. The objective is to develop patient-centric robotics that enhance a customized and empowering healthcare experience as we overcome obstacles and move forward. By putting the patient at the center of their care, these technologies seek to improve everyday well-being in addition to revolutionizing medical procedures [240]. Table 5 depicts an overview of various types of robots used in healthcare.

**Table 5.** Overview of healthcare-related robots.

Robot Type	Applications	Key features	References
Surgical Robots	Minimally invasive surgery Remote surgery Microsurgery	Precision control Enhanced dexterity Integration with imaging technologies	[241]
Rehabilitation Robots	Physical therapy support Gait training Motor function recovery	Adaptive motion Feedback systems	[242]
Telepresence Robots	Remote medical consultations	Realtime communication Mobility	[243]
Robotic Exoskeleton	Mobility support	Assisted walking Muscle support	[244]
Soft Robots	Assisted surgery Endoscopy	Flexibility Biocompatibility	[245]
Nano Robots	Targeted drug delivery Medical imaging	Small scale Precision	[246]

## 7. Biomechanics

As a crucial component of biomedical engineering, biomechanics is leading the charge to transform healthcare by deciphering the complex mechanical dynamics of the human body. Biomechanics, which is based on the combination of biology, physics, and engineering concepts, is an essential lens that helps us understand the physiological details of movement and function. This field explores the forces, structures, and motions that control the biomechanical properties of living things, providing a deep comprehension of the mechanical operations of the body [247].

Computational biomechanics offers an in-silico method for studying the mechanical behavior of biological systems by fusing concepts from biology, medicine, and engineering. Beginning in 1970, the development of sophisticated numerical tools and the quick increase in processing capacity made it possible to apply computational techniques to biomechanical problems. New constitutive laws and codes were created to characterize the mechanical response of both soft and hard tissue [248]. Computational biomechanics has advanced dramatically because of Finite Element (FE) analysis, finding widespread use in a range of clinical domains including brain, heart, and cardiovascular medicine, gastrointestinal, urinary, and musculoskeletal system, as well as at the cellular level. By using medical imaging techniques like computed tomography (CT) and magnetic resonance imaging (MRI), and even with the support of machine learning and artificial intelligence to improve the accuracy in the model geometry, one major advancement has been the introduction of patient-specificity in computational models. Customization offers a precise depiction of each patient’s anatomy and perhaps tissue characteristics, allowing for the prediction of treatment outcomes and/or the identification of the best surgical approaches for that particular patient [249].

A new area of biomechanics called ‘stomach-bariatric computational modelling’ has shown promise in thoroughly analyzing several gastric problems, including gastroesophageal reflux disease and the consequences of bariatric surgery on the stomach’s mechanical response and gastric wall solicitation. A robust and dependable biomechanical characterization combined with FE analysis may prove to be an invaluable and potent clinical instrument, enabling not only the pre-examination of novel surgical techniques and tools but also the comparison of current bariatric interventions, such as the Laparoscopic Sleeve Gastrectomy (LSG) and the Endoscopic Sleeve Gastroplasty (ESG), suggesting creative surgical strategies to prevent complications after the procedure. The computation of the deformation field using the biomechanical model is only the first step in the image registration process for biomechanics-based image registration [250]. The preoperative image must be warped to match the intraoperative organ arrangement during surgery using the computed deformation field. Because this needs to be done intraoperatively, highly efficient picture warping algorithms are required. The prediction of brain deformations based on biomechanics holds great potential for improving surgical management of epilepsy, a persistent neurological condition. Although surgery is ‘arguably the most underutilized of all proven effective therapeutic interventions in the field of medicine,’ it can be curative [251]. The study of biomechanics in both normal and disordered masticatory systems is increasingly reliant on computational models of interactions within the craniomandibular apparatus. Health professionals are frequently faced with assessing the appropriateness, validity, and significance of models that are maybe more recognized to the engineering community. The methods and assumptions in these models might be challenging for people who are not familiar with current procedures in this field [252].

The study of Osseo integrated implant biomechanics has been aided by the application of engineering expertise in dentistry. The biomechanical load on implants has been assessed using several methods, including strain gauge analysis, photo elastic stress analysis, and finite element stress analysis. The study of Osseo integrated implant biomechanics has been aided by the application of engineering expertise in dentistry. Nevertheless, the literature on the effects of various biomechanical variables is equivocal, and the mechanisms underlying biomechanical implant failures remain poorly understood [253].

## 8. Green biomaterials

Natural polymers, which are derived from proteins or polysaccharides, have garnered significant attention in the biomedical field owing to their extensive potential applications. These materials’ chemical stability, structural adaptability, biocompatibility, and great availability make them suitable for a wide range of uses in fields like tissue engineering, medication delivery, and wound healing. Biomaterials that have been extracted from plants or animals have also been modified to enhance their structural characteristics or encourage interactions with surrounding cells and tissues for better in vivo performance. This has led to the development of novel uses for biomaterials, including surface coatings, implantable devices, and controlled drug release [254]. Traditional biomaterials used in biomedicine, like collagen, silk, and gelatin [255], were initially employed in clinical settings in the 1950s and were obtained from natural sources. Even if they have so far had a significant impact on patients’ quality of life, they are constantly being altered to enhance their material characteristics, bioactivities, and appropriateness for therapeutic applications by taking advantage of developments in the disciplines of molecular and cellular biology and polymer chemistry. Additionally, new and modified materials that can stay in close and useful contact with bodily tissues for extended periods of time are being developed as biomaterials grow into new applications like medication delivery, tissue engineering, scaffolds, and bioprinting [256]. Because it contains a wide range of growth hormones, effector chemicals, enzymes, and cellular adhesion motifs that affect gene expression, intracellular signaling, and cell proliferation, natural extracellular matrix (ECM) can function as a porous 3D microenvironment ‘scaffold.’ However, because extracellular matrix (ECM) is so complex and variable, scaffolds utilized in tissue engineering are usually more specifically designed to support certain tasks like cell adhesion or differentiation [257].

**Figure 13.**
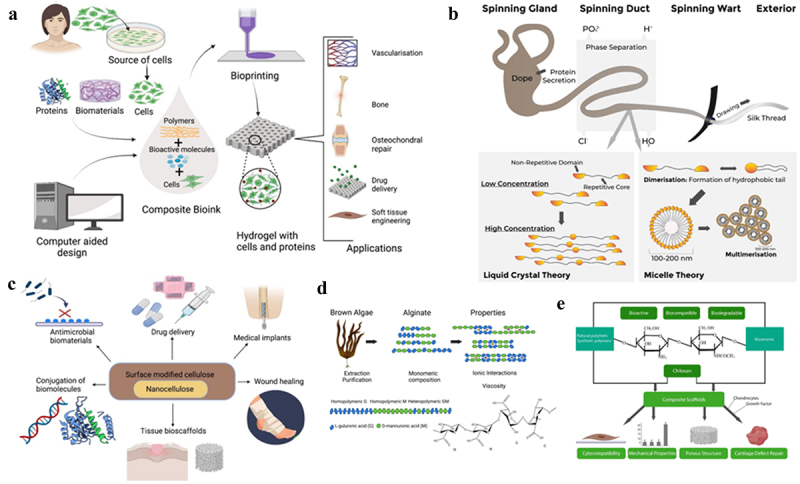
(a) Bioprinting of natural polymers, frequently in combination with cells and/or biomolecules to fine-tune or increase in vivo activity, has potential to provide carefully designed, highly structured materials for tissue and organ engineering applications. (b) silk spinning process (upper level) with liquid crystal and micelle theories of fiber formation (lower panels). (c) Examples of the uses of cellulose as a biomaterial. (d) Alginate is extracted and purified from a wide variety of brown algae. (e) Composition of the chitosan polymer and examples of properties that influence its biomedical uses (adapted with permission from ref [292], copyright, 2021, MDPI).

The textile industry has been using silk, a fibrous protein, for centuries. Arthropods, such as silk ‘worms,’ which are butterflies and moths (order Lepidoptera) and members of the class Arachnida (which includes about 34,000 species of spiders), naturally make it [254]. These creatures use specific endothelium cells to make silk, which is then secreted into the glandular lumen and spun into strands to construct webs, cocoons, nets, and traps. The next step is to harvest and unravel the cocoons to extract the silk threads, of which silk fibroin the primary component protein is particularly useful for applications in tissue engineering, medication delivery, and wound healing. Following the removal of sericin, the fibrils or threads are often dissolved in calcium nitrate, lithium thiocyanate, and 4-methylmorpholine N-oxide before being dialyzed against pure water to remove electrolytes and create silk-based materials for biomedical applications. Before scaffolds or other biomaterials are produced, the finished fibroin solution can be stored at room temperature for a few weeks or at a low temperature for several months [244]. Figure 13 shows the nature based biomaterials and their applications.

The exceptional strength, toughness, weight, flexibility, and extensibility are provided by the nanofibrillar silk structure. It is perfectly suited for biological applications due to its extraordinarily high strength-to-density ratio [258]. Surprisingly, it has been discovered that silk fibers are just as strong as steel and nylon and stronger than Kevlar. Silk fibroins don’t pose a risk for infection, and although some materials haven’t received clinical approval because of their immunogenicity, it’s possible that this is because of the outer sericin layer, since the core silk materials only slightly cause inflammatory or immune responses, whereas sericin can cause allergic and immunological reactions as well as the release of TNF-α. The immunogenic response to silk fibroin is lowered to a level lower than that of collagen or synthetic PLGA when sericin is removed [259]. Enzymatic degradation of silk fibroins causes a gradual loss of mechanical strength in vivo, with nontoxic breakdown products. Overall, few weeks after implantation, silk-based biomaterials can be 100% biodegradable. Pre-clinical studies on silk fibroin in fields including gene therapy and wound healing. When osteoblasts were seeded into scaffolds, silk films functionalized through the chemical binding of RGD domains encouraged the production of bone; in human cell culture, silk and collagen films demonstrated similar capacities to support cell binding, differentiation, and physiological morphology. It has been shown that a silk/chitosan composite film with 40–50% chitosan is a viable matrix for use in artificial skin and wound dressing, and that seeding the film with human adipose-derived stem cells may help repair soft tissue wounds in mice models [260]. One of the hardest known biomaterials is spider silk. Spiridon is one of the large spider silk proteins that are responsible for the arrangement of the material’s mechanical attributes. While spider silks can absorb up to three times as much weight as Kevlar and have been used in biomedicine for centuries, natural fibers usually exhibit lower strength and stiffness than synthetic materials. The ancient Greeks and Romans used spider silk poultices to cover wounds and function as styptics (anti-antihaemorrhagics). However, because they are generated in much smaller amounts and are more difficult to develop in big numbers due to the predatory nature of spiders, they have proven to be more difficult to commercialize than insect (B. mori) silks [261]. Pre-clinical investigations have looked into the potential application of MA silk from the Nephila species in bladder reconstruction. In order to create a mesh that allowed primary human urothelial cell (HUC) adhesion and development in vitro without the need for extra biological stimuli, single silk threads were cross-woven [262].

There are several different sources of polysaccharide biomaterials, which are used in the food, pharmaceutical, biomedical, and cosmetics industries. The biomaterials comprise cellulose, the most prevalent polysaccharide on the planet, alginates, the structural cell wall component of numerous seaweeds, and chitin and its derivative chitosan from crustacean and fungal sources. In addition to certain bacterial sources, plant-based materials including cotton, wood, and other plant sources can also provide cellulose [254]. With an annual production of about 30 billion tonnes of natural cellulose biomass, it is the most abundant biopolymer found in nature. Nanofibre networks constructed of celluloses are chosen for use in biological applications. Bodin et al. [263] compared the mechanical properties of a BNC gel with collagen implants commonly used to treat degenerative meniscal lesions. This boosts the celluloses’ surface area and improves their interactions with polymers and biomaterials. Utilizing the advantages of both cellulose and nanomaterials, nanocelluloses are cellulose extracts made up of structured nanoscale components. Although nanoparticles are known to pose health and environmental hazards, nanocellulose fibers are permanently bonded to the cellulose molecule. The use of nanocellulose in lesion repair, as scaffolds to support cell culture, and in tissue repair/regeneration has advanced significantly in recent years [264]. Zang et al. [265] produced a BNC artificial blood vessel of 100 mm length and 1 mm thickness using Gluconacetobacter xylinum. The mechanical characteristics of collagen implants, which are frequently used to treat degenerative meniscal lesions, were compared with BNC gel by Bodin et al. [263]. Grown to a thickness of 5–15 mm, the BC gel demonstrated a far higher capacity to support loads than collagen, and it could also be shaped into the shape of a meniscus and encourage cell migration. Using a reverse ear mold, a BNC prototype in the shape of an ear was also created, proving that BC could effectively replicate the mechanical characteristics of natural ear cartilage and could be molded to create ear replacements that were tailored to the needs of each patient [266]. Lin et al. [267] is shown in his paper the production of artificial blood vessels is an area of interest (with BNC in particular) due to the mechanical strength and blood biocompatibility of nanocellulose-based materials. In a patient with endocrine neoplasia, the scaffolds were effectively used as prosthetic blood arteries in the carotid artery. Particularly BNC has generated a lot of interest in the field of wound care because it seems to have little cytotoxicity and promote the growth of several human cell types, including human adipose-derived stem cells. Compared to commercial wound dressings, BNC-based biomaterials treated wound beds more quickly to promote tissue regeneration and the development of new capillaries.It is evident that cellulose and nanocellulose have many uses, including medication administration, wound healing, and replacement implants [268]. This is largely because of their biocompatibility, adaptability, and tunable chemical and physical properties. The primary drawback of nanocellulose biomaterials as scaffolds may be their intrinsic non-degradability; hence, their greatest potential may lie in medication delivery and wound healing, particularly when paired with antimicrobial agents for enhanced efficacy [269].

Derived from chitin, the second most abundant natural polymer on Earth, chitosan is a special kind of biopolymer that has garnered a lot of attention lately due to its potential uses in tissue engineering. The exoskeleton of crustaceans, like crabs, and the cell membrane of plants, such fungus hyphae (mushrooms), contain both chitin and chitosan. However, because chitosan is soluble in mildly acidic solutions, it has found widespread use in tissue engineering, ophthalmology, and wound healing/dressing due to its adaptability, biodegradability, and biocompatibility [270]. Because of its well-known antibacterial and antifungal qualities, chitosan is a popular choice for biomedical scaffolds. Although the exact mechanism underlying its antibacterial effect is unknown, its positive charge is thought to interact with microbes’ negatively charged cell surfaces to prevent material uptake and excretion [271]. It has also been observed that low molecular weight chitosan can pierce bacterial cell walls and attach itself to DNA through its protonated amino groups, disrupting vital microbial processes including RNA creation. It has also been demonstrated that chitosan plays a role in apoptosis induction, which affects the direct destruction of cancer cells. Chitosan blocks nerve endings to help coagulate blood and lessen discomfort. These hemostatic characteristics are explained by negatively charged red blood cells being drawn to the protonated amine groups, which causes blood cells to aggregate and form clots, which stop bleeding rapidly [272]. Because of its adaptability and ease of production into 2D films and fibers as well as 3D scaffolds like hydrogels and sponges, chitosan is employed in wound healing. Chitosan’s remarkable mucoadhesive qualities make the substance suitable for use in medication administration applications [273].

Alginates, which are hydrophilic and anionic polymers, are among the most extensively distributed biosynthesized compounds on the planet. They can be found in the cell walls of bacterial species like Pseudomonas and Azotobacter as well as brown algae like Laminaria hyperborea and Macrocystis pyrifera. Alginate gels have been extensively used in many biomedical domains, such as bone and tissue regeneration, wound healing, and the use of model systems for the study of mammalian cell culture [274]. Alginate gels have been used to transport proteins and low molecular weight chemicals over an extended period of time. Proteins can be readily integrated into alginate gels without denaturing them, and the gels prevent the proteins from degrading and enable their regulated release, making alginate a suitable vehicle for the administration of protein-based medications [275]. Alginate gels have been extensively employed in the transportation of proteins and cells for the purpose of tissue and organ engineering or regeneration. Alginate is also one of the biomolecules most frequently utilized in 3D bioprinting because it works well with both extrusion and inkjet printing techniques and because it has characteristics that are comparable to those of natural extracellular matrix (ECM), which may sustain cell growth [276].

## 9. Challenges

Although the combination of engineering and biological sciences has ushered in a new era of innovation in healthcare, it is not without its difficulties. Overcoming obstacles including multidisciplinary communication impediments, legal frameworks, and ethical considerations is essential to the integration of these fields [277]. Advanced medical technology, gene editing, and precision medicine present difficult moral conundrums that must be carefully navigated to ensure fair and responsible application [278]. To maintain standards and protect patient safety, regulatory procedures need to keep up with the quick speed of technical changes. In addition, it is still difficult to develop productive partnerships between engineers and medical specialists, which emphasizes the necessity of strong multidisciplinary communication to convert ground-breaking discoveries into real benefits for patient care [279].

Also, coordinating legal and regulatory frameworks with the quick speed of innovation is an ongoing challenge given the dynamic environment of healthcare technologies. The bounds of medical capabilities are constantly being redefined by advancements in biology and engineering, and legal and ethical frameworks are finding it difficult to keep up. The difficult task of creating regulations that strike a balance between protecting patient rights and privacy and promoting innovation falls to policymakers [1]. Maintaining this delicate balance necessitates ongoing cooperation between researchers, industry stakeholders, and regulatory organizations in order to predict and resolve new issues. Complexity is further increased by the transnational character of scientific collaboration, which makes standardization across national boundaries necessary to provide a unified and morally sound worldwide approach to the use of biomedical engineering in healthcare [280].

Furthermore, difficulties outside of the technological sphere arise when translating state-of-the-art research into useful applications. Overcoming the divide between engineers and medical professionals is still an ongoing challenge. Understanding the vocabulary of each profession is necessary for effective interdisciplinary collaboration, but so is a shared dedication to the overarching objective of better patient outcomes [281]. It is critical to create an environment where professionals in biomedical research and engineering may communicate freely and with respect for one another. Organizations and institutions must aggressively support collaborative efforts, creating a climate in which knowledge can be smoothly incorporated and ideas can flow freely. In order to fully realize the revolutionary potential of engineering in conjunction with biomedical sciences and to make sure that breakthroughs result in real advantages for healthcare globally, it will be imperative to overcome these obstacles [282].

## 10. Future prospects

Medical imaging is poised to become even more accurate, noninvasive, and widely available in the future, driven by rapid technological advancements. Portable imaging devices, quantum imaging, and AI-driven image processing hold promise for enhancing the timing and precision of disease detection. Moreover, integrating imaging modalities with additional diagnostic instruments like proteomics and genomes may enable personalized medicine strategies for targeted therapies. Biomedical sensors, characterized by smaller size, higher sensitivity, and better connectivity, will continue to shape the future of healthcare [11]. The development of wearable and implantable sensors will enable real-time tracking of illness progression, biomarkers, and vital signs, facilitating preventive medicine through remote patient monitoring, early intervention, and personalized healthcare management. Upcoming drug delivery technologies aim to increase patient compliance, minimize adverse effects, and enhance therapeutic efficacy. Stimuli-responsive drug release mechanisms, targeted delivery systems, and nanotechnology-based drug carriers offer precise control over drug dosage and distribution throughout the body. Injectable or implantable drug-release devices hold potential as long-term treatments for chronic illnesses [283]. In the realm of tissue engineering and regenerative medicine, promising areas include tissue healing, organ replacement, and disease modeling. Breakthrough advancements in biomaterials, 3D bioprinting, and biofabrication techniques allow for the creation of complex tissues and organs ex vivo, offering novel approaches to treating congenital problems, traumatic injuries, and degenerative diseases through stem cell therapy, gene editing, and tissue regeneration techniques [224].

Biomedical automation and robotics are advancing by merging robotics, AI, and machine learning to improve assistive technology, rehabilitative treatments, and surgical accuracy. Surgical robots equipped with telemedicine-enabled robotic surgery, autonomous surgical procedures, and augmented reality assistance are poised to revolutionize healthcare delivery, particularly in remote or resource-constrained environments [231]. Prostheses and robotic exoskeletons will enhance mobility and quality of life for individuals with disabilities. Meanwhile, developments in biomechanics will deepen our understanding of musculoskeletal function, human mobility, and injury prevention, enabling ergonomic workplace design, sports performance optimization, and personalized rehabilitation programs through computational models, wearable sensors, and motion analysis technology [284]. Future biomaterials will prioritize biodegradability, biocompatibility, and sustainability, replacing traditional synthetic biomaterials with eco-friendly, bio-derived materials made from renewable resources. This shift toward greener biomaterials, along with bio-inspired designs and self-healing biomaterials, offers creative solutions for tissue engineering, drug delivery, and medical device applications, contributing to a more sustainable future in healthcare. Overall, the convergence of engineering and biological sciences holds immense potential to revolutionize global health outcomes through cutting-edge methods for medical diagnosis, imaging, drug delivery, and regenerative medicine, ultimately transforming patient care with early disease identification, customized treatments, and improved quality of life [285].

## 11. Conclusion

The merger of engineering and biological sciences is resulting in the creation of new paradigms in the field of healthcare technology. The accuracy of medical imaging, the difficulties of drug distribution, and the possibilities of tissue engineering and regenerative medicine are all examples of how these technologies are affecting patient care. Automation, robotics, and biological sensors all contribute to significantly amplifying the transformative effects that are already present. In spite of the challenges that lie ahead, the future appears to be bright considering the promise of AI-driven diagnostics and personalized therapy. It will be necessary for us to make collaborative efforts and take ethical considerations into account as we move forward in order to guarantee that these technologies are available to everyone. An excellent illustration of advancement, the combination of engineering and biological sciences has the potential to raise the bar for medical care in any region of the world.

## Supplementary Material

Graphical abstract one.jpg

## Data Availability

The data analyzed in this study were sourced from publicly available articles in the open literature. All references to the data used, including article titles, authors, and publication sources, are provided in the reference list of this article. Readers and researchers interested in accessing the raw data can refer to the original publications cited herein for retrieval from the respective sources.

## List of Abbreviations

AI: Artificial intelligence
ESI: Electrical Source Imaging
MSI: Magnetic Source Imaging
SPECT: Single- Photon Emission Computed Tomography
MRI: Magnetic resonance imaging
MRS: Magnetic Resonance Spectroscopy
PET: Positron Emission Tomography
EIT: Electrical Impedance Tomography
CT: Computed Tomography
FIB-SEM: Focused Ion Beam – Scanning Electron Microscope
PAI: Photo acoustic Imaging
PPT: Photo Acoustic Tomography
PA: Photo Acoustic
DNA: Deoxyribonucleic Acid
RNA: Ribonucleic Acid
FE: Finite Element
FDA: Food and Drug Administration
SPR: Surface Plasmon Resonance
GNPs: Gold Nanoparticles
GNRs: Gold Nano Rods
HIV: Human Immunodeficiency Virus
PSA: Prostate Specific Antigen
FM: Femtomolar
PM: Pico molar
AgNPs: Silver Nanoparticles
ELISA: Enzyme – Linked Immunoassay
MNPs: Magnetic Nano Particles
SiNPs: Silicon Nano Particles
CEA: Carcinoembryonic Antigen
PtNPs: Platinum Nano Particles
HPV: Human Papilloma Virus
EPCAM: Epithelial Cell Adhesion Molecule
FET: Field Effect Transistor
TET: Thin Film Transistor
SPR: Surface Plasma Resonance
CGM: Continuous Glucose Monitors
MEMS: Micro Electromechanical System
ISR: Intestinal Fluid
LSPR: Localized Surface Plasmon Resonance
MN: Micro Needle
SNP: Single Nucleotide Polymorphism
ECG: Electrocardiogram
PPG: Photo Plethysmography
DDS: Drug Delivery System
SHsp: Small Heat Shock Protein
HSPC: Hydrogenated Soy Phosphatidylcholine
BBB: Blood Brain Barrier
AIDS: Acquired Immunodeficiency Syndrome
CNT: Carbon Nano Tube
TB: Tuberculosis
MI: Myocardial Infraction
CAD: Coronary Artery Disease
MLs: Magneto liposomes
SDDS: Smart Drug Delivery System
LRDD: Light Responsive Drug Delivery
PDT: photodynamic therapy
ROS: Reactive Oxygen Species
MMP: Metalloproteinase
UPA: Urokinase type Plasminogen Activator
DDD: Drug Delivery Develop
RP: Robotic Pill
SC: Stratum Coreneum
TE: Tissue Engineering
TERM: Tissue Engineering and Regenerative Medicine
ECM: Extracellular Matrix
FE: Finite Element
