# Supplementary figures and images for: Engineering with Biomedical Sciences Changing the Horizon of Healthcare-A Review
Source: Bioengineered. 2024 Sep 16;15(1):2401269. doi: 10.1080/21655979.2024.2401269 (PMC11409512; doi:10.1080/21655979.2024.2401269)

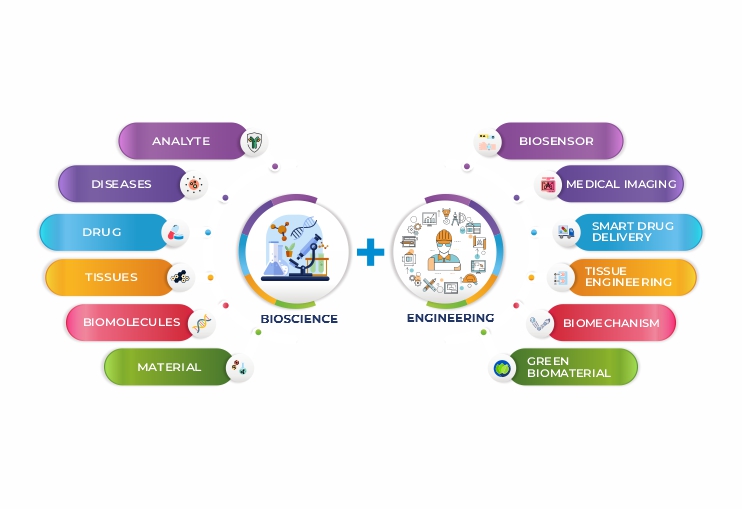

Supplement: Graphical abstract one.jpg [file KBIE_A_2401269_SM9106.jpg]
